# DNA sequence analysis suggests that *cytb-nd1* PCR-RFLP may not be applicable to sandfly species identification throughout the Mediterranean region

**DOI:** 10.1007/s00436-015-4865-5

**Published:** 2016-01-12

**Authors:** Ivonne Pamela Llanes-Acevedo, Carolina Arcones, Rosa Gálvez, Oihane Martin, Rocío Checa, Ana Montoya, Carmen Chicharro, Susana Cruz, Guadalupe Miró, Israel Cruz

**Affiliations:** Servicio de Parasitología, Centro Nacional de Microbiología, Instituto de Salud Carlos III, World Health Organization Collaborating Center for Leishmaniasis, Ctra. Majadahonda-Pozuelo Km2, Majadahonda, 28220 Madrid, Spain; Departamento de Sanidad Animal, Facultad de Veterinaria, Universidad Complutense de Madrid, Avda. Puerta de Hierro s/n, 28040 Madrid, Spain; Neglected Tropical Diseases Programme, Foundation for Innovative New Diagnostics-FIND, Chemin des Mines 9, Campus Biotech, 1202 Geneva, Switzerland

**Keywords:** Mediterranean phlebotomine sandflies, Mitochondrial DNA, PCR-RFLP, *cytb*

## Abstract

Molecular methods are increasingly used for both species identification of sandflies and assessment of their population structure. In general, they are based on DNA sequence analysis of targets previously amplified by PCR. However, this approach requires access to DNA sequence facilities, and in some circumstances, it is time-consuming. Though DNA sequencing provides the most reliable information, other downstream PCR applications are explored to assist in species identification. Thus, it has been recently proposed that the amplification of a DNA region encompassing partially both the *cytochrome-B* (*cytb*) and the *NADH dehydrogenase 1* (*nd1*) genes followed by RFLP analysis with the restriction enzyme *Ase* I allows the rapid identification of the most prevalent species of phlebotomine sandflies in the Mediterranean region. In order to confirm the suitability of this method, we collected, processed, and molecularly analyzed a total of 155 sandflies belonging to four species including *Phlebotomus ariasi*, *P. papatasi*, *P. perniciosus*, and *Sergentomyia minuta* from different regions in Spain. This data set was completed with DNA sequences available at the GenBank for species prevalent in the Mediterranean basin and the Middle East. Additionally, DNA sequences from 13 different phlebotomine species (*P. ariasi*, *P. balcanicus*, *P. caucasicus*, *P. chabaudi*, *P. chadlii*, *P. longicuspis*, *P. neglectus*, *P. papatasi*, *P. perfiliewi*, *P. perniciosus*, *P. riouxi*, *P. sergenti*, and *S. minuta*), from 19 countries, were added to the data set. Overall, our molecular data revealed that this PCR-RFLP method does not provide a unique and specific profile for each phlebotomine species tested. Intraspecific variability and similar RFLP patterns were frequently observed among the species tested. Our data suggest that this method may not be applicable throughout the Mediterranean region as previously proposed. Other molecular approaches like DNA barcoding or phylogenetic analyses would allow a more precise molecular species identification.

**Electronic supplementary material:**

The online version of this article (doi:10.1007/s00436-015-4865-5) contains supplementary material, which is available to authorized users.

## Introduction

Hematophagous females of certain species of phlebotomine sandflies (Diptera: Psychodidae: Phlebotominae) are vectors of human and different animal pathogens worldwide (Pigott et al. 2014). They are responsible for the transmission of diseases caused by protozoans (*Leishmania*), viruses (Phlebovirus, Vesiculovirus, and Orbivirus), and bacteria (*Bartonella bacilliformis*) (Antoniou et al. 2008; Depaquit et al. 2010; Ready 2013; Maroli et al. 2013). (i) In the Mediterranean basin, different species of sandflies are vectors of *Leishmania tropica*, *L. major*, *L. infantum*, and *L. donovani*, the causative agents of leishmaniasis; (ii) the main viral diseases transmitted by phlebotomine sandflies in this region are caused by Phlebovirus (family Bunyaviridae), like the sandfly fever (SF) caused by the SF Sicilian virus and the SF Naples virus, as well as the summer meningitis caused by Toscana virus.

Both leishmaniasis and sandfly-borne viral diseases represent a serious threat to public veterinary health and are considered (re-)emerging infections in the Mediterranean area. Therefore, vector surveillance should be among the pillars of the activities devoted to the design and implementation of effective control measures against these diseases (WHO/EMRO 2008; Depaquit et al. 2010; Charrel et al. 2012; Maroli et al. 2013; Antoniou et al. 2013; Ejov and Dagne 2014). Although fragile in nature, sandflies are able to adapt to a broad variety of environmental conditions including urban or peri-urban settings; the recent leishmaniasis outbreak in Madrid (Spain) is an example of this plasticity (Tarallo et al. 2010; Galvez et al. 2011; Carrillo et al. 2013; Ready 2013; Lisi et al. 2014). Their potential to spread to northern Europe cannot be omitted; the northward spread of leishmaniasis in Italy has been well documented, and the potential establishment of vector species in Germany is a cause of concern (Maroli et al. 2008; Biglino et al. 2010; Fischer et al. 2010; Melaun et al. 2014; Medlock et al. 2014). Due to their public health importance, surveillance of sandfly populations is critical to assess both their geographical distribution and that of the diseases they transmit, as well as routes of introduction to non-endemic areas.

As only some species of sandflies are confirmed vectors, an important aspect of surveillance is the appropriate identification of the obtained specimens. Traditionally, the identification of sandflies at species level has relied on the morphological analysis of anatomical structures such as the pharynx, spermathecae and cibarium of the females, and the terminal genitalia of the males (Killick-Kendrick et al. 1991). This approach requires a high degree of expertise and can be challenging when the specimens are not properly preserved. To contribute to this task, different molecular methods have been proposed as complementary approaches for specimen identification. It has been suggested that the amplification of a DNA target encompassing a fragment of the mitochondrial genes *cytb* and *nd1* and the subsequent digestion of the amplicons with the endonuclease *Ase* I would be a useful tool for the rapid identification of the most common phlebotomine sandflies in the Mediterranean region (Latrofa et al. 2012). However, it is important to take into account that this study was based on specimens collected from a limited geographical area of Italy (Basilicata). In addition, in a recent report, Bounamous et al. (2014) failed to effectively distinguish some phlebotomine species in Algeria, even belonging to different genera (like *P. ariasi* and *S. schwetzi*).

In the present study, we aimed to assess whether the methodology proposed by Latrofa et al. (2012) was also useful for sandfly species identification in specimens from a wider geographical origin, including Spain and other endemic areas, where sandfly-borne diseases are endemic and entomological identification is required for surveillance.

## Material and methods

We used two different approaches to test the PCR-RFLP protocol proposed by Latrofa et al. (2012). The first one consisted on a *wet-lab* PCR-*Ase* I RFLP analysis on selected representative sequences of each species collected in Spain (Assembly 1: phlebotomine sandflies from Spain (*N* = 155), see below); the second approach was an in silico analysis of the DNA sequences of the sandflies from assembly 1 plus a collection of 277 DNA sequences retrieved from the GenBank database belonging to 12 different species of sandflies that were collected by other authors in other Mediterranean and Middle East countries (Assembly 2: DNA sequences retrieved from the GenBank (*N* = 277), see below).

### Assembly 1: phlebotomine sandflies from Spain (*N* = 155)

**Fig. 1:**
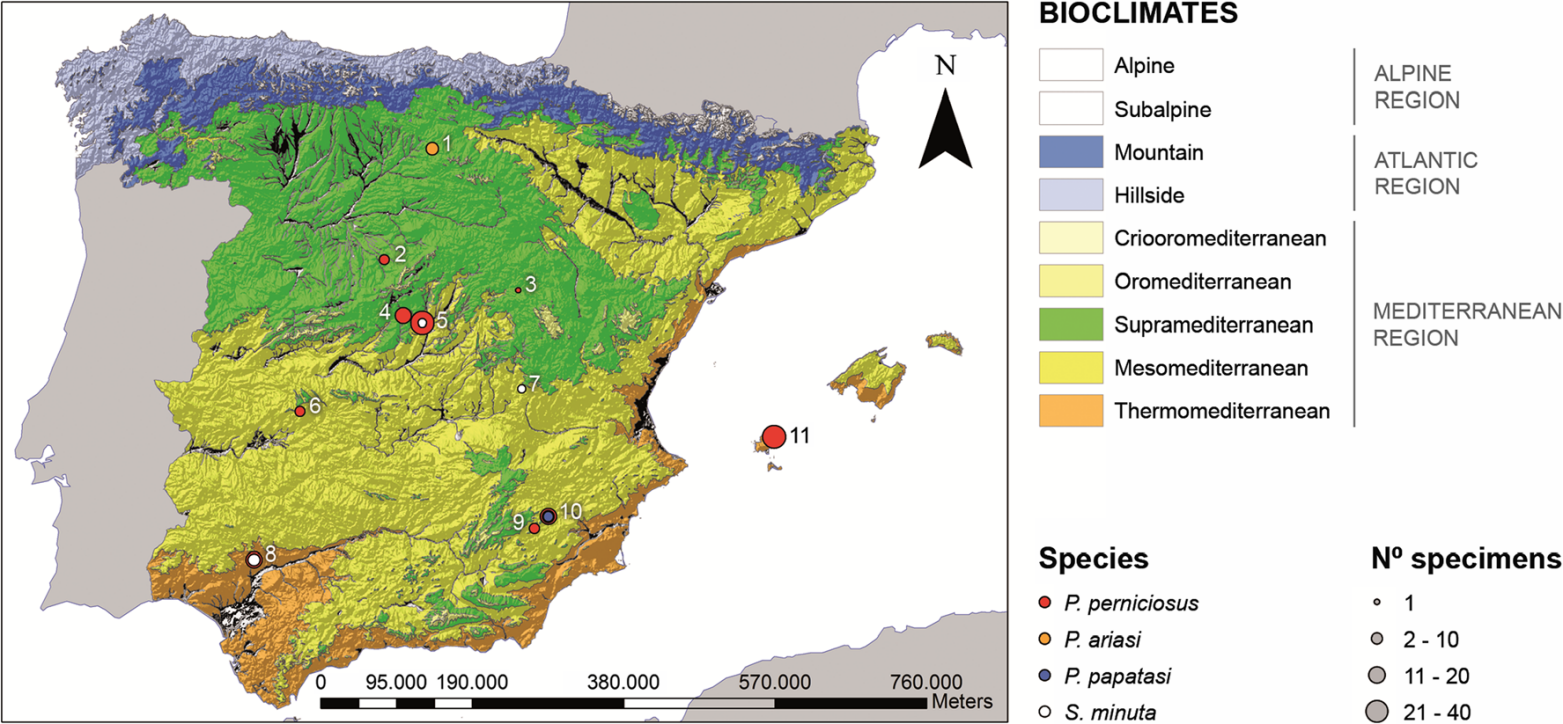
Geocoded surveyed sites and number of sandflies collected shown on a bioclimatic zone map of Spain. Numbers indicate sampling locations: Agés (Burgos) (*1*), Escarabajosa de Cabezas (Segovia) (*2*), Zaorejas (Guadalajara) (*3*), Majadahonda (Madrid) (*4*), Vicálvaro (Madrid) (*5*), Cañamero (Cáceres) (*6*), Buenache de Alarcón (Cuenca) (*7*), Castilblanco de los Arroyos (Sevilla) (*8*), Achivel (Murcia) (*9*), Moratalla (Murcia) (*10*), and San Joan de Labritja (Ibiza) (*11*). *Colored filled circles* indicate phlebotomine species. *Circle size* indicates the number of specimens according to the scale shown

The specimens of this assembly were captured at different sites from mainland Spain and the Balearic Islands between June and October 2013, at different time-points within the sandfly activity season in our region (May–October). The specimens were collected using CDC miniature light traps (John W. Hock, Company, Gainesville, FL) and stored in 70° ethanol at −20 °C until further analyses. Sampling sites were selected according to the different bioclimatic zones in Spain (Fig. 1).

Both male and female were morphologically identified based on their genitalia. The pharyngeal armature and cibarium were also studied in the female specimens. The tip of the abdomen (between segments VI and VII) and head of the females were cutoff and cleared in Mark André medium (Abbonnenc 1972). The specimens were mounted onto glass slides in Hoyer medium (Upton 1993) and identified at species level, according to the taxonomic keys proposed by Gil et al. (1989). Mounted parts of the specimens were kept for future reference. As morphological identification was based on the study of the male genitalia and female head and spermatechae, the remaining of the body was kept at −20 °C for DNA extraction and downstream molecular analyses. A detailed description of sampling locations, species (*P. perniciosus*, *P. ariasi*, *P. papatasi*, and *S. minuta*), and number of specimens included in assembly 1 is presented in Fig. 1 and Table 1.

**Table 1:** Description of sampling locations, species, and number of specimens included in assembly 1 (*N* = 155)

Province	Site	Date (dd/mm/yyyy)	Coordinates	Species	No. of specimens (male/female)
Burgos	Agés	15/08/2013	42.37 N 3.49 W	*P. ariasi*	12 (12/0)
Segovia	Escarabajosa de Cabezas	15/08/2013	41.10 N 4.20 W	*P. perniciosus*	7 (7/0)
Guadalajara	Zaorejas	06/07/2013	39.38 N 5.40 W	*P. perniciosus*	1 (1/0)
Madrid	Majadahonda	08/08/2013	40.48 N 3.91 W	*P. perniciosus*	18 (18/0)
Madrid	Vicálvaro	07/10/2013	40.40 N 3.62 W	*P. perniciosus*	21 (17/4)
Madrid	Vicálvaro	07/10/2013	40.40 N 3.62 W	*S. minuta*	1 (1/0)
Cuenca	Buenache de Alarcón	15/08/2013	39.65 N 2.16 W	*S. minuta*	1 (1/0)
Cáceres	Cañamero	03/08/2013	39.38 N 5.40 W	*P. perniciosus*	3 (1/2)
Murcia	Archivel	23/07/2013	38,07 N 2.00 W	*P. perniciosus*	8 (3/5)
Murcia	Moratalla	23/07/2013	39.02 N 1.49 E	*P. perniciosus*	17 (3/14)
Murcia	Moratalla	23/07/2013	39.02 N 1.49 E	*P. papatasi*	2 (2/0)
Murcia	Moratalla	23/07/2013	39.02 N 1.49 E	*S. minuta*	3 (3/0)
Sevilla	Castilblanco de los Arroyos	24/06/2013	37.69 N 6.00 W	*P. perniciosus*	11 (10/1)
Sevilla	Castilblanco de los Arroyos	24/06/2013	37.69 N 6.00 W	*S. minuta*	10 (10/0)
Ibiza	Sant Joan de Labritja	01/10/2013	39.02 N 1.49 E	*P. perniciosus*	40 (38/2)

Phlebotomine sandflies captured in Spain, June–October 2013

### Assembly 2: DNA sequences retrieved from the GenBank (*N* = 277)

In order to increase our study sample and to complete the picture of representative sandflies in the Mediterranean region beyond the specimens captured in Spain (assembly 1), we retrieved from GenBank a total of 277 DNA sequences corresponding to 13 different phlebotomine species (*P. ariasi*, *P. balcanicus*, *P. caucasicus*, *P. chabaudi*, *P. chadlii*, *P. longicuspis*, *P. neglectus*, *P. papatasi*, *P. perfiliewi*, *P. perniciosus*, *P. riouxi*, *P. sergenti*, and *S. minuta*) containing the *cytb-nd1* sequence encompassed by the primers PhleF and PhleR described by Latrofa et al. (2011). These sequences were identified by BLASTn search using as query different DNA sequences obtained from specimens in assembly 1. Alternatively, taxon-specific key (*Phlebotomus*, *Sergentomyia*) and gene identifier (*cytb, cyt b, cyt-b, cytochrome b*, *cytochrome-b*) were also used to search for candidate sequences. Selected DNA sequences included those from species endemic to the Mediterranean basin (including Italy and Algeria), the Middle East (Afghanistan and Iran), and other regions (Portugal) where the former species are also present. Details on the specimens included in assembly 2 are presented in Table 2. Further details related to these sequences are presented as supplementary material (Supplementary material 1).

**Table 2:** Description of the sampling location, species, and number of specimens included in assembly 2 (*N* = 277), for which *cytb-nd1* sequences were retrieved from the GenBank

Species	Country
(No. of specimens)	(No. of specimens)
*P. perniciosus* (26)	Italy (6), Malta (3), Portugal (6), Spain (1), Tunisia (9), Italy/Malta/Tunisia (1)^a^
*P. longicuspis* (6)	Morocco (1), Tunisia (5)
*P. ariasi* (45)	Algeria (1), France (10), Portugal (8), Spain (26)
*P. papatasi* (31)	Afghanistan (4), Cyprus (2), Egypt (5), Iran (1), Israel (3), Italy (8), Jordan (1), Palestine (3), Syria (2), Turkey (2)
*P. chabaudi* (32)	Algeria (5), Tunisia (27)
*P. neglectus* (6)	Italy (6)
*P. perfiliewi* (26)	Algeria (4), Greece (5), Italy (17)
*P. riouxi* (13)	Algeria (9), Tunisia (4)
*P. sergenti* (57)	Greece (1), Iran (48), Lebanon (1), Morocco (1), Sria (1), Tunisia (4), Turkey (1)
*P. chadlii* (2)	Algeria (2)
*P. balcanicus* (2)	Iran (2)
*P. caucasicus* (25)	Afghanistan (4), Iran (21),
*S. minuta* (6)	Italy (6)

Sandflies were captured from 1997 to2003, according to the information provided by the authors in the sequence identifiers or its associated publication (information available in Supplementary material 1)

^a^The sequence was associated to these three countries by its authors

### DNA extraction and *cytb-nd1* PCR from assembly 1

Genomic DNA was independently extracted from each specimen of assembly 1. Specimens were grinded and processed with the QIAamp DNA Mini Kit (QIAgen, Germany) in accordance with the manufacturer’s instructions. DNA was eluted in 50 μL of PCR-grade water and stored at −20 °C.

The mitochondrial DNA target encompassing the *cytb*-*nd1* region was amplified according to Latrofa et al. (2012) with minor modifications. Two microliters of DNA was used in a 25-μL final volume PCR reaction, including standard reaction buffer 2 mM MgCl_2_, 0.2 mM each dNTP, and 0.7 U of *Thermus* sp. DNA polymerase (Biotools, B&M Labs, Spain), and 15 pmol of each primer PhleF (5′-AAT AAA TTA GGA GGA GTA ATT GC-3′) and PhleR (5′-GCC TCG AWT TCG WTT ATG ATA AAT T-3′) (Sigma-Genosys).

Amplification was performed on a 9800 Fast Thermal Cycler (Applied Biosystems), standard ramp enabled, with the following conditions: initial denaturation of 5 min at 94 °C, 40 cycles at 94 °C for 1 min, 52 °C for 1 min, and 72 °C for 1 min, and final extension at 72 °C for 10 min. All the amplified products were resolved on 2 % agarose gels stained with Pronasafe Nucleic Acid Staining (Laboratorios CONDA, Spain) and visualized under UV light.

### *Ase* I RFLP analysis

Selected representative amplicons obtained at the *cytb*-*nd1*-PCRs from assembly 1 were digested with the endonuclease *Ase* I as described by Latrofa et al. (2012). Digested products were resolved on 2 % agarose gels as described above and their sizes estimated by comparison to a 100-bp DNA ladder (Nippon Genetics, Europe GmbH).

### DNA sequencing

Both strands of the *cytb-nd1* PCR products from assembly 1 were sequenced in duplicate using the same PCR primer set. The Big-Dye Terminator Cycle Sequencing Ready Reaction Kit V3.1 and the automated ABI PRISM 377 DNA sequencer (Applied Biosystems, Foster City, CA) were used. Sequences obtained were analyzed and edited using the software Molecular Evolutionary Genetics Analysis (MEGA) version 5.2 (Tamura et al. 2011).

### Alignment and trimming of the DNA sequences from assembly 1 and assembly 2

DNA sequences generated from the 155 sandfly specimens collected in Spain (assembly 1) and the 277 DNA sequences obtained from the GenBank (assembly 2) were loaded in MEGA 5.2 and aligned using the ClustalW algorithm (Thompson et al. 1994). The sequences were trimmed to the *cytb-nd1* sequence flanked by the primers PhleF and PhleR. A *P. perniciosus* DNA sequence retrieved from the GenBank (Accession Number JF766956) was used as reference sequence to guide both alignment and trimming. Minor editing to complete the 3′-end was conducted in some cases, provided all other specimens of the same species had identical sequence. Primer sequences were added to the end of each sequence to simulate the PCR product.

### In silico *Ase* I RFLP

A virtual *Ase* I RFLP was performed on all trimmed sequences flanked by the PhleF/PhleR primers from assemblies 1 and 2 using the program NEBcutter V2.0 from NEW ENGLAND BioLabs^®^ Inc. (Vincze et al. 2003). which allows to produce theoretical digests with restriction enzymes. Settings for the enzyme *Ase* I included a virtual run of the digested products on a 2 % agarose gel and the full resolution of a 100-bp ladder for DNA sizing, *L* = 70 mm.

## Results

The sequences corresponding to a total of 432 sandfly specimens (assemblies 1 and 2) were tested by the *cytb-nd1*-PCR *Ase* I-RFLP method in silico. In addition, a subset of sequences of assembly 1 was also experimentally assessed. Overall, this study covered 13 sandfly species from five different subgenera: 249 *Larroussius* specimens (152 *P. perniciosus*, 6 *P. longicuspis*, 57 *P. ariasi*, 26 *P. perfiliewi*, 2 *P. chadlii*, 6 *P. neglectus*), 33 *Phlebotomus* specimens (*P. papatasi*), 127 *Paraphlebotomus* specimens (57 *P. sergenti*, 13 *P. riouxi*, 32 *P. chabaudi*, *25 P. caucasicus*), 2 *Adlerius* specimens (*P. balcanicus*), and 21 *Sergentomyia* specimens (*S. minuta*).

### *cytb-nd1-PCR* and experimental *Ase* I RFLP on specimens from assembly 1

**Fig. 2:**
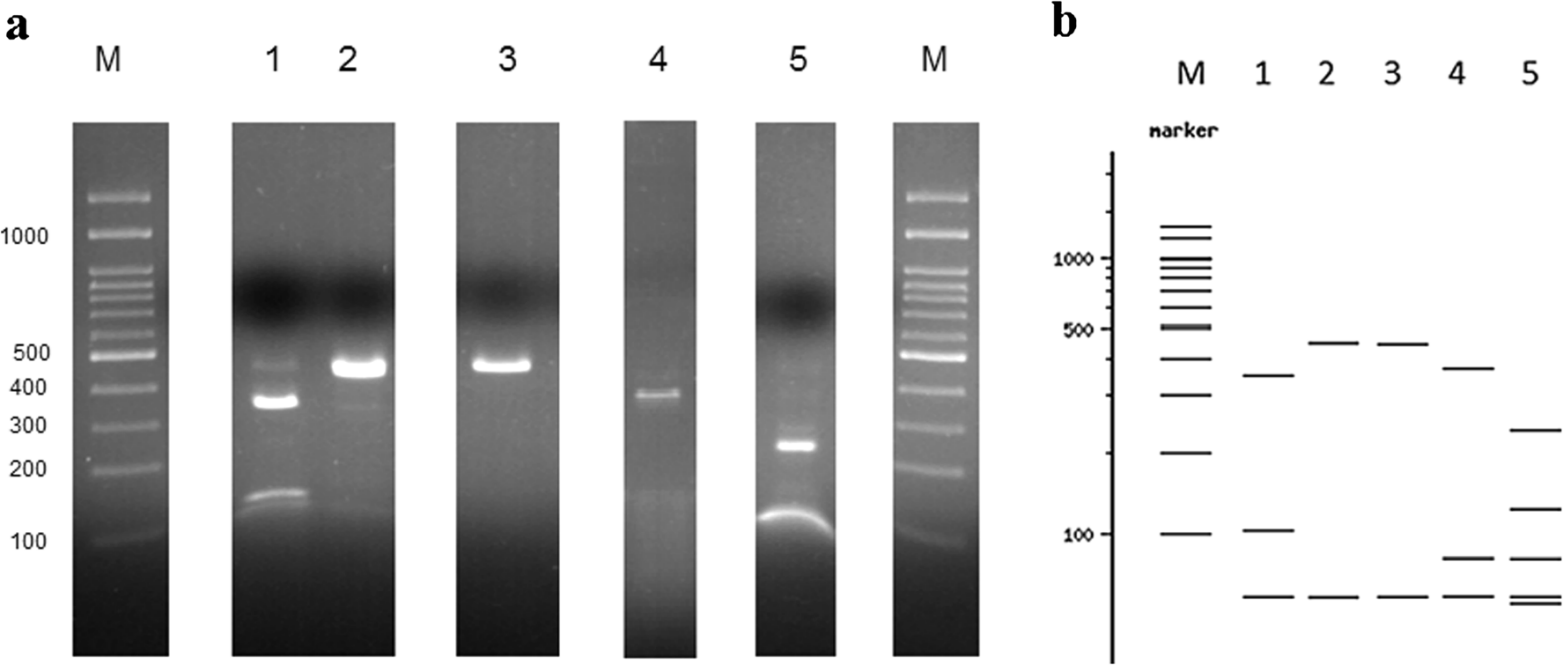
*Ase* I RFLP patterns of the cytb-nd1 PCR products on selected representative sequences of each species collected in Spain (assembly 1). A 100-bp ladder (*lane M*), *P. perniciosus* variant 1 (*lane 1*), *P. perniciosus* variant 2 (*lane 2*), *P. ariasi* variant 1 (*lane 3*), *P. papatasi* (*lane 4*), and *S. minuta* variant-2 (*lane 5*). **a** *Wet-lab* PCR-*Ase* I RFLP analysis and **b** in silico *Ase* I RFLP pattern based on the *cytb-nd1* DNA sequence of the specimens assessed in **a** using NEBcutter V2.0 software

DNA from all specimens in assembly 1 was successfully amplified using the *cytb-nd1-*PCR method. Obtained amplicon sizes ranged from 472 to 482 bp depending on the phlebotomine species considered (Online Resource 2). *Ase* I RFLP analysis was conducted in a limited number of DNA isolates corresponding to representative haplotypes. In all cases, the digested PCR products showed identical restriction patterns to those predicted by the in silico analysis (Fig. 2a, b). Interestingly, *Ase* I RFLP profiles for *P. ariasi-*variant 1 and *P. perniciosus-*variant 2 produced a practically identical pattern comprising bands of 26 and *c*455 bp. On the other hand, *P. perniciosus* variant 1 returned a distinct pattern with two bands of approximately 100 and 350 bp. The *P. papatasi* specimen yielded a band of *c*375 bp, whereas *S. minuta* generated a *c*230-bp band (Fig. 2 and Table 3).

**Table 3:** Summary of patterns obtained by *Ase*I RFLP analysis with the 432 DNA sequences considered in this study

Assigned *Ase* I pattern	Species^a^	Specimens		No. of cuts	Restriction bands (b.p.)	Restriction bands (Latrofa et al. 2012)	Length sequence (b.p.)
I	*P. perniciosus_1*	152	151	2	26, 104, 351	28, 104, 351	481
II	*P. perniciosus_2*		1	1	26, 455		
III	*P. longicuspis_1*	6	5	3	26, 90, 104, 261	ND	
I	*P. longicuspis_2*		1	2	26, 104, 351		
IV	*P. ariasi_1*	57	56	1	26, 454	ND	480
V	*P. ariasi_2*		1	2	26, 178, 276		
VI	*P. papatasi*	33		2	26, 72, 374	28, 72, 374	472
VII	*P. riouxi*	13		1	26, 446	ND	
VII	*P. balcanicus*	2		1	26, 446	ND	
VII	*P. chabaudi_1*	32	31	1	26, 446	ND	
VIII	*P. chabaudi_2*		1	2	26, 57, 389	ND	
IX	*P. neglectus*	6		1	26, 465	28,645	491
X	*P. perfiliewi*	26		3	26, 90, 104, 260	28, 90, 104, 260	480
XI	*P. sergenti_1*	57	52	1	26, 450	ND	476
XII	*P. sergenti_2*		5	2	26, 206, 244		
XIII	*P. chadlii*	2		1	26, 451	ND	477
XI	*P. caucasicus_1*	25	1	1	26, 450	ND	476
XIV	*P. caucasicus_2*		24	2	26, 214, 236		
XV	*S. minuta_1*	21	16	5	17, 26, 40, 72, 91, 236	17, 29, 72, 130, 236	482
XVI	*S. minuta_2*		5	4	17, 26, 72, 131, 236		

Information regarding phlebotomine species number of specimens and predicted size of digested products and length of sequences are provided for direct comparison purposes

*ND* no data

^a^Including species variants in some phlebotomine species analyzed in this study

### Alignment and trimming of *cytb-nd1* DNA sequences from specimens included in assemblies 1 and 2

A final alignment was built with the 155 DNA sequences generated from the assemblies 1 and 2. The alignment (primer sequences included) encompasses the nucleotides at positions 440 to 920 of the *P. perniciosus* sequence used as reference (GenBank Acc no. JF766956) and comprises *cytochrome b* gene (partial cds), *tRNA-Ser* (complete sequence), and *NADH dehydrogenase subunit 1* gene (partial cds). DNA sequence analysis showed a different PCR product size for each species, ranging from 472 to 491 bp. This is mainly due to the sequence length variability of the intergenic mitochondrial DNA spacer-1 (Igs1), lying between *cytb* gene and *tRNA-Ser* (Supplementary material 2).

The 155 DNA sequences from assembly 1 were deposited in GenBank under the following accession numbers: *P. perniciosus* KP685413-KP685538, *P. ariasi* KP685539-KP685550, *P. papatasi* KP702248 and KP702249, and *S. minuta* KP702250-KP702264.

### In silico assessment of *Ase* I RFLP profiles on cytb-nd1 DNA sequences obtained from assemblies 1 and 2

The theoretical in silico *Ase* I RFLP analysis performed on the 432 *cytb-nd1* DNA sequences, comprising 13 different sandfly species, returned 16 (I–XVI) different RFLP profiles. A virtual image of the 16 RFLP patterns is given in Supplementary material 3. Specific details for each specimen are provided in online resource 1. These results were identical to those obtained by experimental *Ase* I RFLP on the selected subset of sandfly specimens from assembly 1 (Fig. 2a, b).

Single, but not necessarily distinctive, RFLP patterns were obtained in all specimens from the following species: *P. papatasi* (pattern VI), *P. neglectus* (IX), *P. perfiliewi* (X), *P. riouxi* (VII), *P. chadlii* (XIII), and *P. balcanicus* (VII). Among all species, only *P. papatasi* gave a unique species-specific pattern that could be unequivocally differentiated. In addition, *P. riouxi* and *P. balcanicus* shared a single RFLP pattern (VII) that was also identical to the variant 1 observed in 31 out of 32 specimens of *P. chabaudi.* The remaining species presented two distinctive patterns each: *P. perniciosus* (151 specimens assigned to pattern I, and the remaining one to pattern II), *P. ariasi* (56 pattern IV, one pattern V), *P. longicuspis* (5 pattern III, one pattern I), *P. chabaudi* (31 pattern VII, one pattern VIII), *P. sergenti* (52 pattern XI, 5 pattern XII), *P. caucasicus* (24 pattern XIV, one pattern XI), and *S. minuta* (16 pattern XV, 5 pattern XVI).

**Fig. 3:**
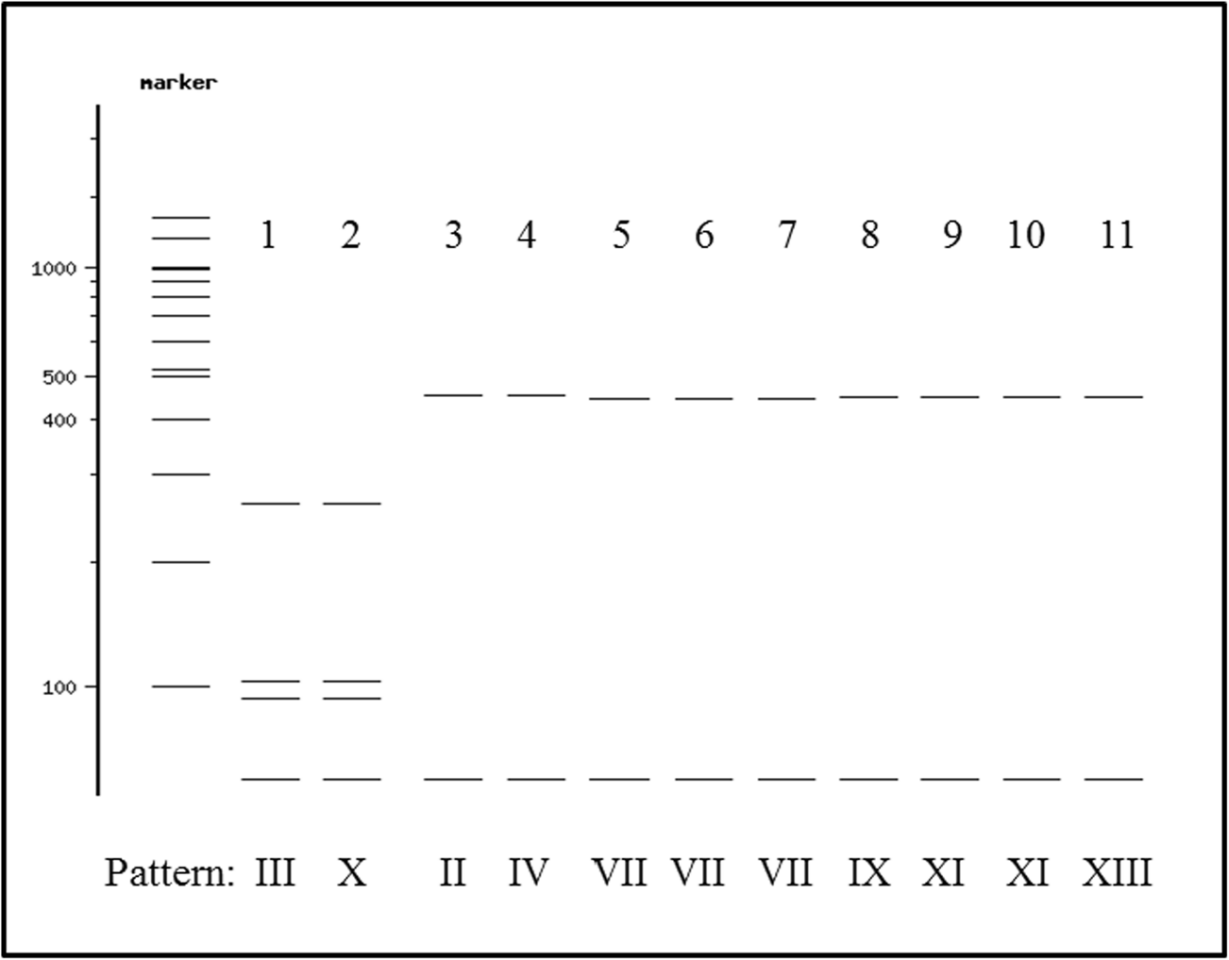
In silico *Ase* I RFLP analysis obtained with NEBcutter V2.0 showing RFLP patterns that are similar or almost undistinguishable. Patterns are indicated in *Roman numerals* at the *bottom of the image*. The virtual run shows the digested products on a theoretical 2 % agarose gel and the full resolution of a 100-bp ladder. *P. longicuspis_1* (*lane 1*), *P. perfiliewi* (*lane 2*), *P. pernicious_2* (*lane 3*), *P. ariasi_1* (*lane 4*), *P. chabaudi_1* (*lane 5*), *P. riouxi* (*lane 6*), *P. balcanicus* (*lane 7*), *P. neglectus* (*lane 8*), *P. sergenti_1* (*lane 9*), *P. caucasicus_1* (*lane 10*), and *P. chadlii* (*lane 11*)

Seven species could not be accurately differentiated due to shared RFLP patterns. Thus, pattern I was common to 151 *P. perniciosus* and one *P. longicuspis*, pattern VII was common to all *P. riouxi*, *P. balcanicus*, and 31 out of 32 *P. chabaudi*, and pattern XI was common to one *P. caucasicus* and 52 out of 57 *P. sergenti*. In addition to this, a number of in silico RFLP patterns were very similar, but not identical. These included pattern III (26, 90, 104, 261 bp) obtained in 5/7 *P. longicuspis* and pattern X (26, 90, 104, 260 bp) from all *P. perfiliewi* analyzed, and also patterns II (26, 455 bp) in 1/152 *P. perniciosus*, IV (26, 454 bp) in 56/57 *P. ariasi*, VII (26, 446 bp) in 31/32 *P. chabaudi*, 13/13 *P. riouxi*, and 2/2 *P. balcanicus*, IX (26, 465 bp) in 6/6 *P. neglectus*, XI (26, 450 bp) in 52/57 *P. sergenti* and 1/25 *P. caucasicus*, and XIII (26, 451 bp) in 2/2 *P. chadlii* (Fig. 3).

## Discussion

Sequence analysis of a *cytb* gene region from 13 different sandfly species provides in silico and experimental evidence demonstrating that the *cytb-nd1* PCR-RFLP assay may not be applicable to phlebotomine species identification throughout the Mediterranean region as initially proposed by other authors. Our data confirm that it is not possible to obtain a unique, distinctive RFLP pattern for each individual sandfly species tested excluding *P. papatasi* and *S. minuta*. We found a significant proportion of species returning identical or almost undistinguishable RFLP profiles. This fact would represent a pitfall for vector incrimination in regions where two coendemic species share the same or almost identical RFLP pattern. This is the case for *P. perniciosus* pattern II and *P. ariasi* pattern IV, both identified in Spanish specimens, being both vector of *L. infantum*. Importantly, this lack of discriminatory power has been previously reported using the same methodology by Bounamous et al. (2014). In that survey, the authors were unable to effectively discriminate among 17 sandfly species present in Algeria, even when a double digestion with *Ase* I and *Mnl* I restriction enzymes was used.

It is important to take into consideration that the study by Latrofa et al. (2012) was restricted to sandfly specimens from the south of Italy. In our subset of Italian specimens (*n* = 6) from the same area, we found similar findings except for *S. minuta*. Therefore, pattern XVI (the one showed by Latrofa et al. 2012) was identified in 5 *S. minuta* specimens, whereas the remaining one (Gen Bank Acc no. JF766981) presented pattern XV, as in all 15 *S. minuta* specimens from Spanish origin.

The similarity of certain RFLP patterns would be particularly problematic for phlebotomine species identification if the patterns correspond to sympatric species. This seems to be the case for Algeria and Tunisia, where *P. longicuspis* and *P. perfiliewi* are coendemic and thought to be potential vectors of *L. infantum* (Ready 2013). Discrimination between both species in these countries would be very difficult to achieve if we obtain patterns III (as in five out of six *P. longicuspis* in this work*)* or X (in all 26 *P. perfiliewi* studied here). In this study, one Moroccan *P. longicuspis* specimen showed PCR-RFLP pattern I, as did the majority of the *P. perniciosus* specimens analyzed. These two species coexist in several countries and are remarkably similar morphologically. In Spain, where only *P. longicuspis* male specimens have been described so far, the presence of *P. longicuspis* and *P. perniciosus* has been questioned (Guernaoui et al. 2005).

On the other hand, the similarity found among patterns II (in 1/152 *P. perniciosus)*, IV (in 56/57 *P. ariasi)*, VII (in 31/32 *P. chabaudi*, *P. riouxi*, and *P. balcanicus*), and IX (*P. neglectus*) would impair the definitive identification due to geographical distribution overlapping between *P. ariasi* and *P. perniciosus*; this would be particularly important in Italy, where *P. ariasi*, *P. perniciosus*, and *P. neglectus* coexist. Although *P. ariasi* and *P. perniciosus* are proven vectors of *L. infantum*, this is not the case of *P. neglectus* (Ready 2013). It is important to point out that *P. riouxi* and *P. chabaudi* are similar from the morphological and taxonomic point of view. Both species are known to occur sympatrically in some geographical regions including Algeria and Tunisia. According to our sequence analysis data, no significant differences were found at the nucleotide level to conclusively consider *P. riouxi* and *P. chabaudi* as independent species, as previously proposed by Bounamous et al. (2014). This notion is also supported by the phylogenetic data obtained with other molecular markers, including *cytb* and *elongation factor 1-α* (Tabbabi et al. 2014). Overall, this information provides molecular and evolutionary evidence suggesting that *P. riouxi* should be considered either as a synonym or as a subspecies of *P. chabaudi*.

Pattern VII was the only profile shared by two different subgenera (*Paraphlebotomus* and *Adlerius*). Thus, it would be useful to analyze the RFLP patterns in other sandfly species belonging to *Adlerius* subgenus endemic to the Mediterranean basin. Similarity between patterns XI (in 52/57 *P sergenti* and in 1/25 *P. caucasicus*) and XIII (*P. chadlii*) would be also a problem regarding sandfly identification because these three phlebotomine species coexist in some geographical areas (see www.sandflycatalog.org)*.* Whereas *P. sergenti* is a proven vector of *L. tropica* (Ready 2013). this potential role has also been suggested for *P. caucasicus* (Artemiev 1978; Killick-Kendrick 1999). However, *P. chadlii* has not been described as suitable vector of the disease to date.

An important contribution of this work is the submission to the GenBank of 155 new DNA sequences for the*cytb-nd1* region from Spanish specimens, including 126 *P. perniciosus* sequences to be added to the single sequence already available at GenBank from this country. Additionally, a total of 15 new *S. minuta* sequences add to the only six previously available sequences, all corresponding to Italian specimens.

Our results demonstrate that neither in Spain nor throughout the Mediterranean region is possible to accurately differentiate phlebotomine sand flies using the *cytb-nd1* PCR-RFLP method. Because sequences from Middle East countries were underrepresented in this study, we cannot draw definitively conclusions on the performance of this method with samples from regions other than the Mediterranean basin. Nevertheless, further work at the subregional level is needed to confirm the extent of these findings. Taking into account the sequence variability observed within the *cytb-nd1* region, we believe that a phylogenetic analysis or a DNA barcoding approach would allow more precise species identification, as it has been proposed by other authors (Esseghir et al. 1997; Bounamous et al. 2008; Kruger et al. 2011; Latrofa et al. 2011). Nevertheless, before these approaches are fully developed, they should be based on a solid morphological identification of the specimens.

## Electronic supplementary material

ESM 1 Excel spreadsheet containing relevant data for each specimen used in the analyses carried out in the present study. (DOCX 96 kb)

ESM 2 Nucleotide alignment of the *cytb-nd1* DNA sequences from representative specimens for each phlebotomine sandfly species studied in this work. The corresponding genes are reported on top of the alignment. Numbers at the bottom indicate nucleotide positions. Numbers at the end of each line indicate the length (in bp) of each sequence. (DOCX 797 kb)

ESM 3 Virtual agarose gel produced with NEBcutter V2.0 showing the sixteen different RFLP patterns obtained from the *cytb-nd1* DNA sequences included in assemblies 1 and 2. Patterns are indicated in Roman numerals. The virtual run shows the digested products on a theoretical 2% agarose gel electrophoresis and the full resolution of a 100 bp ladder molecular weight marker, L=70mm. Pattern I: *P. perniciosus_1*, *P. longicuspis_2*; Pattern II: *P. perniciosus_2*; Pattern III: *P. longicuspis_1*; Pattern IV: *P. ariasi_1*; Pattern V: *P. ariasi_2*; Pattern VI: *P. papatasi*; Pattern VII: *P. riouxi, P. balcanicus, P. chabaudi_1*; Pattern VIII: *P. chabaud_2i*; Pattern IX: *P. neglectus*; Pattern X: *P. perfiliewi*; Pattern XI: *P. sergenti_1*, *P. caucasicus_1*; Pattern XII: *P. sergenti_2*; Pattern XIII: *P. chadlii*; Pattern XIV: *P. caucasicus_2*; Pattern XV: *S. minuta_1*; Pattern XVI: *S. minuta_2*. (DOCX 217 kb)
