# Supplementary material for: DNA sequence analysis suggests that cytb-nd1 PCR-RFLP may not be applicable to sandfly species identification throughout the Mediterranean region
Source: Parasitol Res. 2016 Jan 12;115:1287–95. doi: 10.1007/s00436-015-4865-5 (PMC4759228; doi:10.1007/s00436-015-4865-5)
Supplement: Supplementary file 1 — Excel spreadsheet containing relevant data for each specimen used in the analyses carried out in the present study. (DOCX 96 kb) [file 436_2015_4865_MOESM1_ESM.docx]

| **Assembly** | **Species** | **Collection year** | **Location (Country: Site, Province, or Region)** | **GenBank Acc No.** | **Reference**** | ***cytb-nd1* PCR product (bp)** | **Rectriction bands (bp)predicted** | **RFLP pattern** |
| --- | --- | --- | --- | --- | --- | --- | --- | --- |
| 1 | *Phlebotomus perniciosus* | 2013 | **Spain:** Archivel, Murcia | KP685413 | This work | 481 | 26, 104, 351 | I |
| 1 | *Phlebotomus perniciosus* | 2013 | **Spain:** Archivel, Murcia | KP685414 | This work | 481 | 26, 104, 351 | I |
| 1 | *Phlebotomus perniciosus* | 2013 | **Spain:** Archivel, Murcia | KP685415 | This work | 481 | 26, 104, 351 | I |
| 1 | *Phlebotomus perniciosus* | 2013 | **Spain:** Archivel, Murcia | KP685416 | This work | 481 | 26, 104, 351 | I |
| 1 | *Phlebotomus perniciosus* | 2013 | **Spain:** Archivel, Murcia | KP685417 | This work | 481 | 26, 104, 351 | I |
| 1 | *Phlebotomus perniciosus* | 2013 | **Spain:** Archivel, Murcia | KP685418 | This work | 481 | 26, 104, 351 | I |
| 1 | *Phlebotomus perniciosus* | 2013 | **Spain:** Archivel, Murcia | KP685419 | This work | 481 | 26, 104, 351 | I |
| 1 | *Phlebotomus perniciosus* | 2013 | **Spain:** Archivel, Murcia | KP685420 | This work | 481 | 26, 104, 351 | I |
| 1 | *Phlebotomus perniciosus* | 2013 | **Spain:** Cañamero, Cáceres | KP685421 | This work | 481 | 26, 104, 351 | I |
| 1 | *Phlebotomus perniciosus* | 2013 | **Spain:** Cañamero, Cáceres | KP685422 | This work | 481 | 26, 104, 351 | I |
| 1 | *Phlebotomus perniciosus* | 2013 | **Spain:** Cañamero, Cáceres | KP685423 | This work | 481 | 26, 104, 351 | I |
| 1 | *Phlebotomus perniciosus* | 2013 | **Spain:** Castilblanco de los Arroyos, Sevilla | KP685424 | This work | 481 | 26, 104, 351 | I |
| 1 | *Phlebotomus perniciosus* | 2013 | **Spain:** Castilblanco de los Arroyos, Sevilla | KP685425 | This work | 481 | 26, 104, 351 | I |
| 1 | *Phlebotomus perniciosus* | 2013 | **Spain:** Castilblanco de los Arroyos, Sevilla | KP685426 | This work | 481 | 26, 104, 351 | I |
| 1 | *Phlebotomus perniciosus* | 2013 | **Spain:** Castilblanco de los Arroyos, Sevilla | KP685427 | This work | 481 | 26, 104, 351 | I |
| 1 | *Phlebotomus perniciosus* | 2013 | **Spain:** Castilblanco de los Arroyos, Sevilla | KP685428 | This work | 481 | 26, 104, 351 | I |
| 1 | *Phlebotomus perniciosus* | 2013 | **Spain:** Castilblanco de los Arroyos, Sevilla | KP685429 | This work | 481 | 26, 104, 351 | I |
| 1 | *Phlebotomus perniciosus* | 2013 | **Spain:** Castilblanco de los Arroyos, Sevilla | KP685430 | This work | 481 | 26, 104, 351 | I |
| 1 | *Phlebotomus perniciosus* | 2013 | **Spain:** Castilblanco de los Arroyos, Sevilla | KP685431 | This work | 481 | 26, 104, 351 | I |
| 1 | *Phlebotomus perniciosus* | 2013 | **Spain:** Castilblanco de los Arroyos, Sevilla | KP685432 | This work | 481 | 26, 104, 351 | I |
| 1 | *Phlebotomus perniciosus* | 2013 | **Spain:** Castilblanco de los Arroyos, Sevilla | KP685433 | This work | 481 | 26, 104, 351 | I |
| 1 | *Phlebotomus perniciosus* | 2013 | **Spain:** Castilblanco de los Arroyos, Sevilla | KP685434 | This work | 481 | 26, 104, 351 | I |
| 1 | *Phlebotomus perniciosus* | 2013 | **Spain:** Majadahonda, Madrid | KP685435 | This work | 481 | 26, 104, 351 | I |
| 1 | *Phlebotomus perniciosus* | 2013 | **Spain:** Majadahonda, Madrid | KP685436 | This work | 481 | 26, 104, 351 | I |
| 1 | *Phlebotomus perniciosus* | 2013 | **Spain:** Majadahonda, Madrid | KP685437 | This work | 481 | 26, 104, 351 | I |
| 1 | *Phlebotomus perniciosus* | 2013 | **Spain:** Majadahonda, Madrid | KP685438 | This work | 481 | 26, 104, 351 | I |
| 1 | *Phlebotomus perniciosus* | 2013 | **Spain:** Majadahonda, Madrid | KP685439 | This work | 481 | 26, 104, 351 | I |
| 1 | *Phlebotomus perniciosus* | 2013 | **Spain:** Majadahonda, Madrid | KP685440 | This work | 481 | 26, 104, 351 | I |
| 1 | *Phlebotomus perniciosus* | 2013 | **Spain:** Majadahonda, Madrid | KP685441 | This work | 481 | 26, 104, 351 | I |
| 1 | *Phlebotomus perniciosus* | 2013 | **Spain:** Majadahonda, Madrid | KP685442 | This work | 481 | 26, 104, 351 | I |
| 1 | *Phlebotomus perniciosus* | 2013 | **Spain:** Majadahonda, Madrid | KP685443 | This work | 481 | 26, 104, 351 | I |
| 1 | *Phlebotomus perniciosus* | 2013 | **Spain:** Majadahonda, Madrid | KP685444 | This work | 481 | 26, 104, 351 | I |
| 1 | *Phlebotomus perniciosus* | 2013 | **Spain:** Majadahonda, Madrid | KP685445 | This work | 481 | 26, 104, 351 | I |
| 1 | *Phlebotomus perniciosus* | 2013 | **Spain:** Majadahonda, Madrid | KP685446 | This work | 481 | 26, 104, 351 | I |
| 1 | *Phlebotomus perniciosus* | 2013 | **Spain:** Majadahonda, Madrid | KP685447 | This work | 481 | 26, 104, 351 | I |
| 1 | *Phlebotomus perniciosus* | 2013 | **Spain:** Majadahonda, Madrid | KP685448 | This work | 481 | 26, 104, 351 | I |
| 1 | *Phlebotomus perniciosus* | 2013 | **Spain:** Majadahonda, Madrid | KP685449 | This work | 481 | 26, 104, 351 | I |
| 1 | *Phlebotomus perniciosus* | 2013 | **Spain:** Majadahonda, Madrid | KP685450 | This work | 481 | 26, 104, 351 | I |
| 1 | *Phlebotomus perniciosus* | 2013 | **Spain:** Majadahonda, Madrid | KP685451 | This work | 481 | 26, 104, 351 | I |
| 1 | *Phlebotomus perniciosus* | 2013 | **Spain:** Majadahonda, Madrid | KP685452 | This work | 481 | 26, 104, 351 | I |
| 1 | *Phlebotomus perniciosus* | 2013 | **Spain:** Escarabajosa de Cabezas, Segovia | KP685453 | This work | 481 | 26, 104, 351 | I |
| 1 | *Phlebotomus perniciosus* | 2013 | **Spain:** Escarabajosa de Cabezas, Segovia | KP685454 | This work | 481 | 26, 104, 351 | I |
| 1 | *Phlebotomus perniciosus* | 2013 | **Spain:** Escarabajosa de Cabezas, Segovia | KP685455 | This work | 481 | 26, 104, 351 | I |
| 1 | *Phlebotomus perniciosus* | 2013 | **Spain:** Escarabajosa de Cabezas, Segovia | KP685456 | This work | 481 | 26, 104, 351 | I |
| 1 | *Phlebotomus perniciosus* | 2013 | **Spain:** Escarabajosa de Cabezas, Segovia | KP685457 | This work | 481 | 26, 104, 351 | I |
| 1 | *Phlebotomus perniciosus* | 2013 | **Spain:** Escarabajosa de Cabezas, Segovia | KP685458 | This work | 481 | 26, 104, 351 | I |
| 1 | *Phlebotomus perniciosus* | 2013 | **Spain:** Escarabajosa de Cabezas, Segovia | KP685459 | This work | 481 | 26, 104, 351 | I |
| 1 | *Phlebotomus perniciosus* | 2013 | **Spain:** Vicálvaro, Madrid | KP685460 | This work | 481 | 26, 104, 351 | I |
| 1 | *Phlebotomus perniciosus* | 2013 | **Spain:** Vicálvaro, Madrid | KP685461 | This work | 481 | 26, 104, 351 | I |
| 1 | *Phlebotomus perniciosus* | 2013 | **Spain:** Vicálvaro, Madrid | KP685462 | This work | 481 | 26, 104, 351 | I |
| 1 | *Phlebotomus perniciosus* | 2013 | **Spain:** Vicálvaro, Madrid | KP685463 | This work | 481 | 26, 104, 351 | I |
| 1 | *Phlebotomus perniciosus* | 2013 | **Spain:** Vicálvaro, Madrid | KP685464 | This work | 481 | 26, 104, 351 | I |
| 1 | *Phlebotomus perniciosus* | 2013 | **Spain:** Vicálvaro, Madrid | KP685465 | This work | 481 | 26, 455 | II |
| 1 | *Phlebotomus perniciosus* | 2013 | **Spain:** Vicálvaro, Madrid | KP685466 | This work | 481 | 26, 104, 351 | I |
| 1 | *Phlebotomus perniciosus* | 2013 | **Spain:** Vicálvaro, Madrid | KP685467 | This work | 481 | 26, 104, 351 | I |
| 1 | *Phlebotomus perniciosus* | 2013 | **Spain:** Vicálvaro, Madrid | KP685468 | This work | 481 | 26, 104, 351 | I |
| 1 | *Phlebotomus perniciosus* | 2013 | **Spain:** Vicálvaro, Madrid | KP685469 | This work | 481 | 26, 104, 351 | I |
| 1 | *Phlebotomus perniciosus* | 2013 | **Spain:** Vicálvaro, Madrid | KP685470 | This work | 481 | 26, 104, 351 | I |
| 1 | *Phlebotomus perniciosus* | 2013 | **Spain:** Vicálvaro, Madrid | KP685471 | This work | 481 | 26, 104, 351 | I |
| 1 | *Phlebotomus perniciosus* | 2013 | **Spain:** Vicálvaro, Madrid | KP685472 | This work | 481 | 26, 104, 351 | I |
| 1 | *Phlebotomus perniciosus* | 2013 | **Spain:** Vicálvaro, Madrid | KP685473 | This work | 481 | 26, 104, 351 | I |
| 1 | *Phlebotomus perniciosus* | 2013 | **Spain:** Vicálvaro, Madrid | KP685474 | This work | 481 | 26, 104, 351 | I |
| 1 | *Phlebotomus perniciosus* | 2013 | **Spain:** Vicálvaro, Madrid | KP685475 | This work | 481 | 26, 104, 351 | I |
| 1 | *Phlebotomus perniciosus* | 2013 | **Spain:** Vicálvaro, Madrid | KP685476 | This work | 481 | 26, 104, 351 | I |
| 1 | *Phlebotomus perniciosus* | 2013 | **Spain:** Vicálvaro, Madrid | KP685477 | This work | 481 | 26, 104, 351 | I |
| 1 | *Phlebotomus perniciosus* | 2013 | **Spain:** Vicálvaro, Madrid | KP685478 | This work | 481 | 26, 104, 351 | I |
| 1 | *Phlebotomus perniciosus* | 2013 | **Spain:** Vicálvaro, Madrid | KP685479 | This work | 481 | 26, 104, 351 | I |
| 1 | *Phlebotomus perniciosus* | 2013 | **Spain:** Vicálvaro, Madrid | KP685480 | This work | 481 | 26, 104, 351 | I |
| 1 | *Phlebotomus perniciosus* | 2013 | **Spain:** Sant Joan de Labritja, Ibiza | KP685481 | This work | 481 | 26, 104, 351 | I |
| 1 | *Phlebotomus perniciosus* | 2013 | **Spain:** Sant Joan de Labritja, Ibiza | KP685482 | This work | 481 | 26, 104, 351 | I |
| 1 | *Phlebotomus perniciosus* | 2013 | **Spain:** Sant Joan de Labritja, Ibiza | KP685483 | This work | 481 | 26, 104, 351 | I |
| 1 | *Phlebotomus perniciosus* | 2013 | **Spain:** Sant Joan de Labritja, Ibiza | KP685484 | This work | 481 | 26, 104, 351 | I |
| 1 | *Phlebotomus perniciosus* | 2013 | **Spain:** Sant Joan de Labritja, Ibiza | KP685485 | This work | 481 | 26, 104, 351 | I |
| 1 | *Phlebotomus perniciosus* | 2013 | **Spain:** Sant Joan de Labritja, Ibiza | KP685486 | This work | 481 | 26, 104, 351 | I |
| 1 | *Phlebotomus perniciosus* | 2013 | **Spain:** Sant Joan de Labritja, Ibiza | KP685487 | This work | 481 | 26, 104, 351 | I |
| 1 | *Phlebotomus perniciosus* | 2013 | **Spain:** Sant Joan de Labritja, Ibiza | KP685488 | This work | 481 | 26, 104, 351 | I |
| 1 | *Phlebotomus perniciosus* | 2013 | **Spain:** Sant Joan de Labritja, Ibiza | KP685489 | This work | 481 | 26, 104, 351 | I |
| 1 | *Phlebotomus perniciosus* | 2013 | **Spain:** Sant Joan de Labritja, Ibiza | KP685490 | This work | 481 | 26, 104, 351 | I |
| 1 | *Phlebotomus perniciosus* | 2013 | **Spain:** Sant Joan de Labritja, Ibiza | KP685491 | This work | 481 | 26, 104, 351 | I |
| 1 | *Phlebotomus perniciosus* | 2013 | **Spain:** Sant Joan de Labritja, Ibiza | KP685492 | This work | 481 | 26, 104, 351 | I |
| 1 | *Phlebotomus perniciosus* | 2013 | **Spain:** Sant Joan de Labritja, Ibiza | KP685493 | This work | 481 | 26, 104, 351 | I |
| 1 | *Phlebotomus perniciosus* | 2013 | **Spain:** Sant Joan de Labritja, Ibiza | KP685494 | This work | 481 | 26, 104, 351 | I |
| 1 | *Phlebotomus perniciosus* | 2013 | **Spain:** Sant Joan de Labritja, Ibiza | KP685495 | This work | 481 | 26, 104, 351 | I |
| 1 | *Phlebotomus perniciosus* | 2013 | **Spain:** Sant Joan de Labritja, Ibiza | KP685496 | This work | 481 | 26, 104, 351 | I |
| 1 | *Phlebotomus perniciosus* | 2013 | **Spain:** Sant Joan de Labritja, Ibiza | KP685497 | This work | 481 | 26, 104, 351 | I |
| 1 | *Phlebotomus perniciosus* | 2013 | **Spain:** Sant Joan de Labritja, Ibiza | KP685498 | This work | 481 | 26, 104, 351 | I |
| 1 | *Phlebotomus perniciosus* | 2013 | **Spain:** Sant Joan de Labritja, Ibiza | KP685499 | This work | 481 | 26, 104, 351 | I |
| 1 | *Phlebotomus perniciosus* | 2013 | **Spain:** Sant Joan de Labritja, Ibiza | KP685500 | This work | 481 | 26, 104, 351 | I |
| 1 | *Phlebotomus perniciosus* | 2013 | **Spain:** Sant Joan de Labritja, Ibiza | KP685501 | This work | 481 | 26, 104, 351 | I |
| 1 | *Phlebotomus perniciosus* | 2013 | **Spain:** Sant Joan de Labritja, Ibiza | KP685502 | This work | 481 | 26, 104, 351 | I |
| 1 | *Phlebotomus perniciosus* | 2013 | **Spain:** Sant Joan de Labritja, Ibiza | KP685503 | This work | 481 | 26, 104, 351 | I |
| 1 | *Phlebotomus perniciosus* | 2013 | **Spain:** Sant Joan de Labritja, Ibiza | KP685504 | This work | 481 | 26, 104, 351 | I |
| 1 | *Phlebotomus perniciosus* | 2013 | **Spain:** Sant Joan de Labritja, Ibiza | KP685505 | This work | 481 | 26, 104, 351 | I |
| 1 | *Phlebotomus perniciosus* | 2013 | **Spain:** Sant Joan de Labritja, Ibiza | KP685506 | This work | 481 | 26, 104, 351 | I |
| 1 | *Phlebotomus perniciosus* | 2013 | **Spain:** Sant Joan de Labritja, Ibiza | KP685507 | This work | 481 | 26, 104, 351 | I |
| 1 | *Phlebotomus perniciosus* | 2013 | **Spain:** Sant Joan de Labritja, Ibiza | KP685508 | This work | 481 | 26, 104, 351 | I |
| 1 | *Phlebotomus perniciosus* | 2013 | **Spain:** Sant Joan de Labritja, Ibiza | KP685509 | This work | 481 | 26, 104, 351 | I |
| 1 | *Phlebotomus perniciosus* | 2013 | **Spain:** Sant Joan de Labritja, Ibiza | KP685510 | This work | 481 | 26, 104, 351 | I |
| 1 | *Phlebotomus perniciosus* | 2013 | **Spain:** Sant Joan de Labritja, Ibiza | KP685511 | This work | 481 | 26, 104, 351 | I |
| 1 | *Phlebotomus perniciosus* | 2013 | **Spain:** Sant Joan de Labritja, Ibiza | KP685512 | This work | 481 | 26, 104, 351 | I |
| 1 | *Phlebotomus perniciosus* | 2013 | **Spain:** Sant Joan de Labritja, Ibiza | KP685513 | This work | 481 | 26, 104, 351 | I |
| 1 | *Phlebotomus perniciosus* | 2013 | **Spain:** Sant Joan de Labritja, Ibiza | KP685514 | This work | 481 | 26, 104, 351 | I |
| 1 | *Phlebotomus perniciosus* | 2013 | **Spain:** Sant Joan de Labritja, Ibiza | KP685515 | This work | 481 | 26, 104, 351 | I |
| 1 | *Phlebotomus perniciosus* | 2013 | **Spain:** Sant Joan de Labritja, Ibiza | KP685516 | This work | 481 | 26, 104, 351 | I |
| 1 | *Phlebotomus perniciosus* | 2013 | **Spain:** Sant Joan de Labritja, Ibiza | KP685517 | This work | 481 | 26, 104, 351 | I |
| 1 | *Phlebotomus perniciosus* | 2013 | **Spain:** Sant Joan de Labritja, Ibiza | KP685518 | This work | 481 | 26, 104, 351 | I |
| 1 | *Phlebotomus perniciosus* | 2013 | **Spain:** Sant Joan de Labritja, Ibiza | KP685519 | This work | 481 | 26, 104, 351 | I |
| 1 | *Phlebotomus perniciosus* | 2013 | **Spain:** Sant Joan de Labritja, Ibiza | KP685520 | This work | 481 | 26, 104, 351 | I |
| 1 | *Phlebotomus perniciosus* | 2013 | **Spain:** Moratalla, Murcia | KP685521 | This work | 481 | 26, 104, 351 | I |
| 1 | *Phlebotomus perniciosus* | 2013 | **Spain:** Moratalla, Murcia | KP685522 | This work | 481 | 26, 104, 351 | I |
| 1 | *Phlebotomus perniciosus* | 2013 | **Spain:** Moratalla, Murcia | KP685523 | This work | 481 | 26, 104, 351 | I |
| 1 | *Phlebotomus perniciosus* | 2013 | **Spain:** Moratalla, Murcia | KP685524 | This work | 481 | 26, 104, 351 | I |
| 1 | *Phlebotomus perniciosus* | 2013 | **Spain:** Moratalla, Murcia | KP685525 | This work | 481 | 26, 104, 351 | I |
| 1 | *Phlebotomus perniciosus* | 2013 | **Spain:** Moratalla, Murcia | KP685526 | This work | 481 | 26, 104, 351 | I |
| 1 | *Phlebotomus perniciosus* | 2013 | **Spain:** Moratalla, Murcia | KP685527 | This work | 481 | 26, 104, 351 | I |
| 1 | *Phlebotomus perniciosus* | 2013 | **Spain:** Moratalla, Murcia | KP685528 | This work | 481 | 26, 104, 351 | I |
| 1 | *Phlebotomus perniciosus* | 2013 | **Spain:** Moratalla, Murcia | KP685529 | This work | 481 | 26, 104, 351 | I |
| 1 | *Phlebotomus perniciosus* | 2013 | **Spain:** Moratalla, Murcia | KP685530 | This work | 481 | 26, 104, 351 | I |
| 1 | *Phlebotomus perniciosus* | 2013 | **Spain:** Moratalla, Murcia | KP685531 | This work | 481 | 26, 104, 351 | I |
| 1 | *Phlebotomus perniciosus* | 2013 | **Spain:** Moratalla, Murcia | KP685532 | This work | 481 | 26, 104, 351 | I |
| 1 | *Phlebotomus perniciosus* | 2013 | **Spain:** Moratalla, Murcia | KP685533 | This work | 481 | 26, 104, 351 | I |
| 1 | *Phlebotomus perniciosus* | 2013 | **Spain:** Moratalla, Murcia | KP685534 | This work | 481 | 26, 104, 351 | I |
| 1 | *Phlebotomus perniciosus* | 2013 | **Spain:** Moratalla, Murcia | KP685535 | This work | 481 | 26, 104, 351 | I |
| 1 | *Phlebotomus perniciosus* | 2013 | **Spain:** Moratalla, Murcia | KP685536 | This work | 481 | 26, 104, 351 | I |
| 1 | *Phlebotomus perniciosus* | 2013 | **Spain:** Moratalla, Murcia | KP685537 | This work | 481 | 26, 104, 351 | I |
| 1 | *Phlebotomus perniciosus* | 2013 | **Spain:** Zaorejas, Guadalajara | KP685538 | This work | 481 | 26, 104, 351 | I |
| 1 | *Phlebotomus ariasi* | 2013 | **Spain:** Agés, Burgos | KP685539 | This work | 480 | 26, 454 | IV |
| 1 | *Phlebotomus ariasi* | 2013 | **Spain:** Agés, Burgos | KP685540 | This work | 480 | 26, 454 | IV |
| 1 | *Phlebotomus ariasi* | 2013 | **Spain:** Agés, Burgos | KP685541 | This work | 480 | 26, 454 | IV |
| 1 | *Phlebotomus ariasi* | 2013 | **Spain:** Agés, Burgos | KP685542 | This work | 480 | 26, 454 | IV |
| 1 | *Phlebotomus ariasi* | 2013 | **Spain:** Agés, Burgos | KP685543 | This work | 480 | 26, 454 | IV |
| 1 | *Phlebotomus ariasi* | 2013 | **Spain:** Agés, Burgos | KP685544 | This work | 480 | 26, 454 | IV |
| 1 | *Phlebotomus ariasi* | 2013 | **Spain:** Agés, Burgos | KP685545 | This work | 480 | 26, 454 | IV |
| 1 | *Phlebotomus ariasi* | 2013 | **Spain:** Agés, Burgos | KP685546 | This work | 480 | 26, 454 | IV |
| 1 | *Phlebotomus ariasi* | 2013 | **Spain:** Agés, Burgos | KP685547 | This work | 480 | 26, 454 | IV |
| 1 | *Phlebotomus ariasi* | 2013 | **Spain:** Agés, Burgos | KP685548 | This work | 480 | 26, 454 | IV |
| 1 | *Phlebotomus ariasi* | 2013 | **Spain:** Agés, Burgos | KP685549 | This work | 480 | 26, 454 | IV |
| 1 | *Phlebotomus ariasi* | 2013 | **Spain:** Agés, Burgos | KP685550 | This work | 480 | 26, 454 | IV |
| 1 | *Phlebotomus papatasi* | 2013 | **Spain:** Moratalla, Murcia | KP702248 | This work | 472 | 26, 72, 374 | VI |
| 1 | *Phlebotomus papatasi* | 2013 | **Spain:** Moratalla, Murcia | KP702249 | This work | 472 | 26, 72, 374 | VI |
| 1 | *Sergentomyia minuta* | 2013 | **Spain:** Buenache de Alarcón, Cuenca | KP702250 | This work | 482 | 17, 26, 40, 72, 91, 236 | XV |
| 1 | *Sergentomyia minuta* | 2013 | **Spain:** Castilblanco de los Arroyos, Sevilla | KP702251 | This work | 482 | 17, 26, 40, 72, 91, 236 | XV |
| 1 | *Sergentomyia minuta* | 2013 | **Spain:** Castilblanco de los Arroyos, Sevilla | KP702252 | This work | 482 | 17, 26, 40, 72, 91, 236 | XV |
| 1 | *Sergentomyia minuta* | 2013 | **Spain:** Castilblanco de los Arroyos, Sevilla | KP702253 | This work | 482 | 17, 26, 40, 72, 91, 236 | XV |
| 1 | *Sergentomyia minuta* | 2013 | **Spain:** Castilblanco de los Arroyos, Sevilla | KP702254 | This work | 482 | 17, 26, 40, 72, 91, 236 | XV |
| 1 | *Sergentomyia minuta* | 2013 | **Spain:** Castilblanco de los Arroyos, Sevilla | KP702255 | This work | 482 | 17, 26, 40, 72, 91, 236 | XV |
| 1 | *Sergentomyia minuta* | 2013 | **Spain:** Castilblanco de los Arroyos, Sevilla | KP702256 | This work | 482 | 17, 26, 40, 72, 91, 236 | XV |
| 1 | *Sergentomyia minuta* | 2013 | **Spain:** Castilblanco de los Arroyos, Sevilla | KP702257 | This work | 482 | 17, 26, 40, 72, 91, 236 | XV |
| 1 | *Sergentomyia minuta* | 2013 | **Spain:** Castilblanco de los Arroyos, Sevilla | KP702258 | This work | 482 | 17, 26, 40, 72, 91, 236 | XV |
| 1 | *Sergentomyia minuta* | 2013 | **Spain:** Castilblanco de los Arroyos, Sevilla | KP702259 | This work | 482 | 17, 26, 40, 72, 91, 236 | XV |
| 1 | *Sergentomyia minuta* | 2013 | **Spain:** Castilblanco de los Arroyos, Sevilla | KP702260 | This work | 482 | 17, 26, 40, 72, 91, 236 | XV |
| 1 | *Sergentomyia minuta* | 2013 | **Spain:** Vicálvaro, Madrid | KP702261 | This work | 482 | 17, 26, 40, 72, 91, 236 | XV |
| 1 | *Sergentomyia minuta* | 2013 | **Spain:** Moratalla, Murcia | KP702262 | This work | 482 | 17, 26, 40, 72, 91, 236 | XV |
| 1 | *Sergentomyia minuta* | 2013 | **Spain:** Moratalla, Murcia | KP702263 | This work | 482 | 17, 26, 40, 72, 91, 236 | XV |
| 1 | *Sergentomyia minuta* | 2013 | **Spain:** Moratalla, Murcia | KP702264 | This work | 482 | 17, 26, 40, 72, 91, 236 | XV |
| 2 | *Phlebotomus perniciosus* | 2008, 2009 | **Italy:** Putignano, Bari | JF766954 | 21635869 | 481 | 26, 104, 351 | I |
| 2 | *Phlebotomus perniciosus* | 2008, 2009 | **Italy:** Putignano, Bari | JF766955 | 21635869 | 481 | 26, 104, 351 | I |
| 2 | *Phlebotomus perniciosus* | 2008, 2009 | **Italy:** Putignano, Bari | JF766956 | 21635869 | 481 | 26, 104, 351 | I |
| 2 | *Phlebotomus perniciosus* | 2008, 2009 | **Italy:** Putignano, Bari | JF766957 | 21635869 | 481 | 26, 104, 351 | I |
| 2 | *Phlebotomus perniciosus* | 2008, 2009 | **Italy:** Putignano, Bari | JF766958 | 21635869 | 481 | 26, 104, 351 | I |
| 2 | *Phlebotomus perniciosus* | 2011-s | **Malta?** | JN036724 | 22897038* | 481 | 26, 104, 351 | I |
| 2 | *Phlebotomus perniciosus* | 2011-s | **Malta?** | JN036725 | 22897038* | 481 | 26, 104, 351 | I |
| 2 | *Phlebotomus perniciosus* | 2011-s | **Malta?** | JN036727 | 22897038* | 481 | 26, 104, 351 | I |
| 2 | *Phlebotomus perniciosus* | 2011-s | **Tunisia?** | JN036728 | 22897038* | 481 | 26, 104, 351 | I |
| 2 | *Phlebotomus perniciosus* | 2011-s | **Portugal:** Lisboa | JN036729 | 22897038 | 481 | 26, 104, 351 | I |
| 2 | *Phlebotomus perniciosus* | 2011-s | **Portugal:** Lisboa? | JN036730 | 22897038* | 481 | 26, 104, 351 | I |
| 2 | *Phlebotomus perniciosus* | 2011-s | **Portugal:** Lisboa? | JN036731 | 22897038* | 481 | 26, 104, 351 | I |
| 2 | *Phlebotomus perniciosus* | 2011-s | **Portugal:** Lisboa? | JN036732 | 22897038* | 481 | 26, 104, 351 | I |
| 2 | *Phlebotomus perniciosus* | 2011-s | **Portugal:** Lisboa? | JN036733 | 22897038* | 481 | 26, 104, 351 | I |
| 2 | *Phlebotomus perniciosus* | 2011-s | **Portugal:** Lisboa? | JN036734 | 22897038* | 481 | 26, 104, 351 | I |
| 2 | *Phlebotomus perniciosus* | 2011-s | **Spain:** Orgiva, Granada | JN036735 | 22897038 | 481 | 26, 104, 351 | I |
| 2 | *Phlebotomus perniciosus* | 2011-s | **Italy:** Gargano, Foggia | JN036737 | 22897038 | 481 | 26, 104, 351 | I |
| 2 | *Phlebotomus perniciosus* | 2011-s | **Tunisia:** Mahdia | JN036738 | 22897038 | 481 | 26, 104, 351 | I |
| 2 | *Phlebotomus perniciosus* | 2011-s | **Tunisia:** Monastir | JN036742 | 22897038 | 481 | 26, 104, 351 | I |
| 2 | *Phlebotomus perniciosus* | 2011-s | **Tunisia:** Mahdia? | JN036743 | 22897038* | 481 | 26, 104, 351 | I |
| 2 | *Phlebotomus perniciosus* | 2011-s | **Tunisia:** Tataouine | JN036744 | 22897038 | 481 | 26, 104, 351 | I |
| 2 | *Phlebotomus perniciosus* | 2011-s | **Tunisia:** Tataouine | JN036745 | 22897038 | 481 | 26, 104, 351 | I |
| 2 | *Phlebotomus perniciosus* | 2011-s | **Tunisia:** Tataouine | JN036748 | 22897038 | 481 | 26, 104, 351 | I |
| 2 | *Phlebotomus perniciosus* | 2011-s | **Tunisia:** Tataouine | JN036754 | 22897038 | 481 | 26, 104, 351 | I |
| 2 | *Phlebotomus perniciosus* | 2011-s | **Tunisia:** Tataouine | JN036755 | 22897038 | 481 | 26, 104, 351 | I |
| 2 | *Phlebotomus perniciosus* | 1997-s | **Italy, Malta, Tunisia** | U94472 | 9272439 | 481 | 26, 104, 351 | I |
| 2 | *Phlebotomus longicuspis* | 2010-s | **Morocco:** | HM131126 | 20451525 | 481 | 26, 104, 351 | I |
| 2 | *Phlebotomus longicuspis* | 2011-s | **Tunisia:** Tataouine | JN036757 | 22897038 | 481 | 26, 90, 104, 261 | III |
| 2 | *Phlebotomus longicuspis* | 2011-s | **Tunisia:** Monastir | JN036758 | 22897038 | 481 | 26, 90, 104, 261 | III |
| 2 | *Phlebotomus longicuspis* | 2011-s | **Tunisia:** Monastir | JN036759 | 22897038 | 481 | 26, 90, 104, 261 | III |
| 2 | *Phlebotomus longicuspis* | 2011-s | **Tunisia:** Monastir? | JN036760 | 22897038* | 481 | 26, 90, 104, 261 | III |
| 2 | *Phlebotomus longicuspis* | 2011-s | **Tunisia:** Monastir | JN036761 | 22897038 | 481 | 26, 90, 104, 261 | III |
| 2 | *Phlebotomus ariasi* | 2002, 2003 | **Spain:** Andalucia | HM131081 | 20451525 | 480 | 26, 454 | IV |
| 2 | *Phlebotomus ariasi* | 2002 | **Spain:** Sant Jaume de Llierca, Girona | HM131082 | 20451525 | 480 | 26, 454 | IV |
| 2 | *Phlebotomus ariasi* | 2003 | **Spain:** Benamahoma, Cádiz | HM131083 | 20451525 | 480 | 26, 454 | IV |
| 2 | *Phlebotomus ariasi* | 2003 | **Spain:** Benamahoma, Cádiz | HM131084 | 20451525 | 480 | 26, 454 | IV |
| 2 | *Phlebotomus ariasi* | 2002 | **Spain:** Trévelez, Granada | HM131085 | 20451525 | 480 | 26, 454 | IV |
| 2 | *Phlebotomus ariasi* | 1997 | **Portugal:** Nagozelo do Douro, São João da Pesqueira | HM131086 | 20451525 | 480 | 26, 454 | IV |
| 2 | *Phlebotomus ariasi* | 1997 | **Portugal:** Nagozelo do Douro, São João da Pesqueira | HM131087 | 20451525 | 480 | 26, 454 | IV |
| 2 | *Phlebotomus ariasi* | 1997 | **Portugal:** Nagozelo do Douro, São João da Pesqueira | HM131088 | 20451525 | 480 | 26, 454 | IV |
| 2 | *Phlebotomus ariasi* | 1997 | **Portugal:** Nagozelo do Douro, São João da Pesqueira | HM131089 | 20451525 | 480 | 26, 454 | IV |
| 2 | *Phlebotomus ariasi* | 1989, 1992, 2008, 2009 | **Portugal:** | HM131090 | 20451525 | 480 | 26, 454 | IV |
| 2 | *Phlebotomus ariasi* | 2002 | **Spain:** Sant Jaume de Llierca, Girona | HM131091 | 20451525 | 480 | 26, 454 | IV |
| 2 | *Phlebotomus ariasi* | 1995 | **Spain:** Aracena, Huelva | HM131092 | 20451525 | 480 | 26, 454 | IV |
| 2 | *Phlebotomus ariasi* | 2008 | **Spain:** Madrid | HM131093 | 20451525 | 480 | 26, 454 | IV |
| 2 | *Phlebotomus ariasi* | 2008 | **Spain:** Paredes de Buitrago, Madrid | HM131094 | 20451525 | 480 | 26, 454 | IV |
| 2 | *Phlebotomus ariasi* | 2008 | **Spain**: (several sites), Madrid | HM131095 | 20451525 | 480 | 26, 454 | IV |
| 2 | *Phlebotomus ariasi* | 2008 | **Spain:** Paredes de Buitrago, Madrid | HM131096 | 20451525 | 480 | 26, 454 | IV |
| 2 | *Phlebotomus ariasi* | 2008 | **Spain**: (several sites), Madrid | HM131097 | 20451525 | 480 | 26, 454 | IV |
| 2 | *Phlebotomus ariasi* | 2008 | **Spain**: Santa María de la Alameda, Madrid | HM131098 | 20451525 | 480 | 26, 454 | IV |
| 2 | *Phlebotomus ariasi* | 1995 | **Spain**: Rio Tinto, Huelva | HM131099 | 20451525 | 480 | 26, 454 | IV |
| 2 | *Phlebotomus ariasi* | 2007 | **France**: Sumène, Gard | HM131100 | 20451525 | 480 | 26, 454 | IV |
| 2 | *Phlebotomus ariasi* | 2007 | **France**: Sumène, Gard | HM131101 | 20451525 | 480 | 26, 454 | IV |
| 2 | *Phlebotomus ariasi* | 2002, 2008 | **Spain**: (several sites) | HM131102 | 20451525 | 480 | 26, 454 | IV |
| 2 | *Phlebotomus ariasi* | 1998 | **France**: Roquebrune, Hérault | HM131103 | 20451525 | 480 | 26, 454 | IV |
| 2 | *Phlebotomus ariasi* | 1998 | **France**: L' Aumède, Gard | HM131104 | 20451525 | 480 | 26, 454 | IV |
| 2 | *Phlebotomus ariasi* | 1998 | **France:** Mas-Saint-Loup, Gard | HM131105 | 20451525 | 480 | 26, 454 | IV |
| 2 | *Phlebotomus ariasi* | 1998, 2007 | **France:** (several sites) | HM131106 | 20451525 | 480 | 26, 454 | IV |
| 2 | *Phlebotomus ariasi* | 2008 | **Spain:** Gualchos, Granada | HM131107 | 20451525 | 480 | 26, 454 | IV |
| 2 | *Phlebotomus ariasi* | 1992 | **Portugal:** Freixo de Espada à Cinta, Braganza | HM131108 | 20451525 | 480 | 26, 454 | IV |
| 2 | *Phlebotomus ariasi* | 2007 | **France:** La Valentine, Bouches du Rhône | HM131109 | 20451525 | 480 | 26, 454 | IV |
| 2 | *Phlebotomus ariasi* | 1998 | **France**: Roquebrune, Hérault | HM131110 | 20451525 | 480 | 26, 454 | IV |
| 2 | *Phlebotomus ariasi* | 1998 | **France:** Mas-Saint-Loup, Gard | HM131111 | 20451525 | 480 | 26, 454 | IV |
| 2 | *Phlebotomus ariasi* | 1998 | **France**: L' Aumède, Gard | HM131112 | 20451525 | 480 | 26, 454 | IV |
| 2 | *Phlebotomus ariasi* | 2002 | **Spain:** Valdemalmusa, Huelva | HM131113 | 20451525 | 480 | 26, 454 | IV |
| 2 | *Phlebotomus ariasi* | 2008 | **Spain**: Santa María de la Alameda, Madrid | HM131114 | 20451525 | 480 | 26, 454 | IV |
| 2 | *Phlebotomus ariasi* | 2002 | **Spain:** Sant Jaume de Llierca, Girona | HM131115 | 20451525 | 480 | 26, 454 | IV |
| 2 | *Phlebotomus ariasi* | 2002, 2003, 2006, 2008 | **Spain**: (several sites) | HM131116 | 20451525 | 480 | 26, 454 | IV |
| 2 | *Phlebotomus ariasi* | 2008 | **Spain:** Güejar Sierra, Granada | HM131117 | 20451525 | 480 | 26, 178, 276 | V |
| 2 | *Phlebotomus ariasi* | 2006 | **Spain:** Busquistar, Granada | HM131118 | 20451525 | 480 | 26, 454 | IV |
| 2 | *Phlebotomus ariasi* | 2002 | **Spain:** Cástaras, Granada | HM131119 | 20451525 | 480 | 26, 454 | IV |
| 2 | *Phlebotomus ariasi* | 2008 | **Spain:** Gualchos, Granada | HM131120 | 20451525 | 480 | 26, 454 | IV |
| 2 | *Phlebotomus ariasi* | 1992 | **Portugal:** Freixo de Espada à Cinta, Braganza | HM131121 | 20451525 | 480 | 26, 454 | IV |
| 2 | *Phlebotomus ariasi* | 2008 | **Spain:** Las Sabinas, Granada | HM131122 | 20451525 | 480 | 26, 454 | IV |
| 2 | *Phlebotomus ariasi* | 2008 | **Spain:** Hoya Pedraza, Granada | HM131123 | 20451525 | 480 | 26, 454 | IV |
| 2 | *Phlebotomus ariasi* | 1992 | **Portugal:** Freixo de Espada à Cinta, Braganza | HM131124 | 20451525 | 480 | 26, 454 | IV |
| 2 | *Phlebotomus ariasi* | 2007 | **Algeria:** Aurès | HM131125 | 20451525 | 480 | 26, 454 | IV |
| 2 | *Phlebotomus papatasi* | 2001 | **Iran:** Khorzoogh, Isfahan | AY378318 | 14651648 | 472 | 26, 72, 374 | VI |
| 2 | *Phlebotomus papatasi* | 2006-s | **Egypt:** Kafr ash shaykh | DQ381816 | 17897368 | 472 | 26, 72, 374 | VI |
| 2 | *Phlebotomus papatasi* | 2006-s | **Egypt:** Alexandria | DQ381817 | 17897368 | 472 | 26, 72, 374 | VI |
| 2 | *Phlebotomus papatasi* | 2006-s | **Egypt:** | DQ381818 | 17897368 | 472 | 26, 72, 374 | VI |
| 2 | *Phlebotomus papatasi* | 2006-s | **Egypt?:** | DQ381819 | 17897368 | 472 | 26, 72, 374 | VI |
| 2 | *Phlebotomus papatasi* | 2006-s | **Egypt?:** | DQ381820 | 17897368 | 472 | 26, 72, 374 | VI |
| 2 | *Phlebotomus papatasi* | 2003 | **Israel:** Jordan Valley | DQ381821 | 17897368 | 472 | 26, 72, 374 | VI |
| 2 | *Phlebotomus papatasi* | 2003 | **Israel:** Jordan Valley | DQ381822 | 17897368 | 472 | 26, 72, 374 | VI |
| 2 | *Phlebotomus papatasi* | 2003 | **Israel:** Jordan Valley | DQ381823 | 17897368 | 472 | 26, 72, 374 | VI |
| 2 | *Phlebotomus papatasi* | 2006-s | **Palestine:** | DQ381824 | 17897368 | 472 | 26, 72, 374 | VI |
| 2 | *Phlebotomus papatasi* | 2006-s | **Palestine:** | DQ381826 | 17897368 | 472 | 26, 72, 374 | VI |
| 2 | *Phlebotomus papatasi* | 2006-s | **Palestine:** | DQ381827 | 17897368 | 472 | 26, 72, 374 | VI |
| 2 | *Phlebotomus papatasi* | 2006-s | **Jordan:** | DQ381828 | 17897368 | 472 | 26, 72, 374 | VI |
| 2 | *Phlebotomus papatasi* | 2006-s | **Italy:** | DQ381829 | 17897368 | 472 | 26, 72, 374 | VI |
| 2 | *Phlebotomus papatasi* | 2006-s | **Italy:** | DQ381830 | 17897368 | 472 | 26, 72, 374 | VI |
| 2 | *Phlebotomus papatasi* | 2006-s | **Italy:** | DQ381831 | 17897368 | 472 | 26, 72, 374 | VI |
| 2 | *Phlebotomus papatasi* | 2006-s | **Syria:** | DQ381832 | 17897368 | 472 | 26, 72, 374 | VI |
| 2 | *Phlebotomus papatasi* | 2006-s | **Syria:** | DQ381833 | 17897368 | 472 | 26, 72, 374 | VI |
| 2 | *Phlebotomus papatasi* | 2006-s | **Turkey:** | DQ381834 | 17897368 | 472 | 26, 72, 374 | VI |
| 2 | *Phlebotomus papatasi* | 2006-s | **Turkey:** | DQ381835 | 17897368 | 472 | 26, 72, 374 | VI |
| 2 | *Phlebotomus papatasi* | 2007 | **Afghanistan:** Feyzabad, Badakhshan | HM803194 | 21396252 | 472 | 26, 72, 374 | VI |
| 2 | *Phlebotomus papatasi* | 2009 | **Afghanistan:** Kondoz, Kondoz | HM803210 | 21396252 | 472 | 26, 72, 374 | VI |
| 2 | *Phlebotomus papatasi* | 2009 | **Afghanistan:** Ghowrmach, Badghis | HM803211 | 21396252 | 472 | 26, 72, 374 | VI |
| 2 | *Phlebotomus papatasi* | 2009 | **Afghanistan:** Mazar-e Sharif, Balkh | HM803212 | 21396252 | 472 | 26, 72, 374 | VI |
| 2 | *Phlebotomus papatasi* | 2009 | **Italy:** Bari | HM992926 | 20738865 | 472 | 26, 72, 374 | VI |
| 2 | *Phlebotomus papatasi* | 2009 | **Italy:** Bari | HM992927 | 20738865 | 472 | 26, 72, 374 | VI |
| 2 | *Phlebotomus papatasi* | 2008, 2009 | **Italy:** Putignano, Bari | JF766973 | 21635869 | 472 | 26, 72, 374 | VI |
| 2 | *Phlebotomus papatasi* | 2008, 2009 | **Italy:** Putignano, Bari | JF766974 | 21635869 | 472 | 26, 72, 374 | VI |
| 2 | *Phlebotomus papatasi* | 2008, 2009 | **Italy:** Putignano, Bari | JF766975 | 21635869 | 472 | 26, 72, 374 | VI |
| 2 | *Phlebotomus papatasi* | 1997-s | **Cyprus, Tunisia:** | U94475 | 9272439 | 472 | 26, 72, 374 | VI |
| 2 | *Phlebotomus papatasi* | 2006-s | **Cyprus, Tunisia, Syria:** | DQ381815 | 17897368 | 472 | 26, 72, 374 | VI |
| 2 | *Phlebotomus chabaudi* | 2013-s | **Tunisia:** Ghomrassen, Tataouine | KC433541 | 25171607 | 472 | 26, 446 | VII |
| 2 | *Phlebotomus chabaudi* | 2013-s | **Tunisia:** Ghomrassen, Tataouine | KC433542 | 25171607 | 472 | 26, 446 | VII |
| 2 | *Phlebotomus chabaudi* | 2013-s | **Tunisia:** Ghomrassen, Tataouine | KC433543 | 25171607 | 472 | 26, 446 | VII |
| 2 | *Phlebotomus chabaudi* | 2013-s | **Tunisia:** Ghomrassen, Tataouine | KC433544 | 25171607 | 472 | 26, 446 | VII |
| 2 | *Phlebotomus chabaudi* | 2013-s | **Tunisia:** Ghomrassen, Tataouine | KC433545 | 25171607 | 472 | 26, 446 | VII |
| 2 | *Phlebotomus chabaudi* | 2013-s | **Tunisia:** Ghomrassen, Tataouine | KC433546 | 25171607 | 472 | 26, 446 | VII |
| 2 | *Phlebotomus chabaudi* | 2013-s | **Tunisia:** Ghomrassen, Tataouine | KC433548 | 25171607 | 472 | 26, 446 | VII |
| 2 | *Phlebotomus chabaudi* | 2013-s | **Tunisia:** Ghomrassen, Tataouine | KC433547 | 25171607 | 472 | 26, 446 | VII |
| 2 | *Phlebotomus chabaudi* | 2005 | **Tunisia:** Moknine, Monastir | EU935814 | 19202763 | 472 | 26, 446 | VII |
| 2 | *Phlebotomus chabaudi* | 2005 | **Tunisia:** Sidi Ameur, Monastir | EU935806 | 19202763 | 472 | 26, 446 | VII |
| 2 | *Phlebotomus chabaudi* | 2005 | **Tunisia:** Sidi Ameur, Monastir | EU935807 | 19202763 | 472 | 26, 446 | VII |
| 2 | *Phlebotomus chabaudi* | 2004 | **Tunisia:** Souassi, Mahdia | EU935796 | 19202763 | 472 | 26, 446 | VII |
| 2 | *Phlebotomus chabaudi* | 2005 | **Tunisia:** Sayada, Monastir | EU935798 | 19202763 | 472 | 26, 446 | VII |
| 2 | *Phlebotomus chabaudi* | 2005 | **Tunisia:** Sayada, Monastir | EU935799 | 19202763 | 472 | 26, 446 | VII |
| 2 | *Phlebotomus chabaudi* | 2005 | **Tunisia:** Sayada, Monastir | EU935800 | 19202763 | 472 | 26, 446 | VII |
| 2 | *Phlebotomus chabaudi* | 2005 | **Tunisia:** Skanes, Monastir | EU935801 | 19202763 | 472 | 26, 446 | VII |
| 2 | *Phlebotomus chabaudi* | 2005 | **Tunisia:** Skanes, Monastir | EU935802 | 19202763 | 472 | 26, 446 | VII |
| 2 | *Phlebotomus chabaudi* | 2005 | **Tunisia:** Skanes, Monastir | EU935803 | 19202763 | 472 | 26, 446 | VII |
| 2 | *Phlebotomus chabaudi* | 2005 | **Tunisia:** Sidi Ameur, Monastir | EU935808 | 19202763 | 472 | 26, 446 | VII |
| 2 | *Phlebotomus chabaudi* | 2005 | **Tunisia:** Sidi Ameur, Monastir | EU935809 | 19202763 | 472 | 26, 446 | VII |
| 2 | *Phlebotomus chabaudi* | 2005 | **Tunisia:** M. Kamel, Monastir | EU935810 | 19202763 | 472 | 26, 446 | VII |
| 2 | *Phlebotomus chabaudi* | 2005 | **Tunisia:** Benbla, Monastir | EU935811 | 19202763 | 472 | 26, 446 | VII |
| 2 | *Phlebotomus chabaudi* | 2005 | **Tunisia:** Moknine, Monastir | EU935812 | 19202763 | 472 | 26, 446 | VII |
| 2 | *Phlebotomus chabaudi* | 2005 | **Tunisia:** Moknine, Monastir | EU935813 | 19202763 | 472 | 26, 446 | VII |
| 2 | *Phlebotomus chabaudi* | 2006 | **Algeria:** Menaa, Aurès | EU935791 | 19202763 | 472 | 26, 446 | VII |
| 2 | *Phlebotomus chabaudi* | 2007 | **Algeria:** Menaa, Aurès | EU935792 | 19202763 | 472 | 26, 446 | VII |
| 2 | *Phlebotomus chabaudi* | 2007 | **Algeria:** Menaa, Aurès | EU935793 | 19202763 | 472 | 26, 446 | VII |
| 2 | *Phlebotomus chabaudi* | 2007 | **Algeria:** Ain-Zaatout, Aurès | EU935794 | 19202763 | 472 | 26, 446 | VII |
| 2 | *Phlebotomus chabaudi* | 2005 | **Tunisia:** Teboulba, Monastir | EU935805 | 19202763 | 472 | 26, 446 | VII |
| 2 | *Phlebotomus chabaudi* | 2005 | **Tunisia:** Teboulba, Monastir | EU935804 | 19202763 | 472 | 26, 57, 389 | VIII |
| 2 | *Phlebotomus chabaudi* | 2004 | **Tunisia:** Souassi, Mahdia | EU935797 | 19202763 | 472 | 26, 446 | VII |
| 2 | *Phlebotomus chabaudi* | 2007 | **Algeria:** Ain-Zaatout, Aurès | EU935795 | 19202763 | 472 | 26, 446 | VII |
| 2 | *Phlebotomus neglectus* | 2008, 2009 | **Italy:** Putignano, Bari | JF766959 | 21635869 | 491 | 26, 465 | IX |
| 2 | *Phlebotomus neglectus* | 2008, 2009 | **Italy:** Putignano, Bari | JF766960 | 21635869 | 491 | 26, 465 | IX |
| 2 | *Phlebotomus neglectus* | 2008, 2009 | **Italy:** Putignano, Bari | JF766961 | 21635869 | 491 | 26, 465 | IX |
| 2 | *Phlebotomus neglectus* | 2008, 2009 | **Italy:** Putignano, Bari | JF766963 | 21635869 | 491 | 26, 465 | IX |
| 2 | *Phlebotomus neglectus* | 2008, 2009 | **Italy:** Putignano, Bari | JF766964 | 21635869 | 491 | 26, 465 | IX |
| 2 | *Phlebotomus neglectus* | 2008, 2009 | **Italy:** Putignano, Bari | JF766962 | 21635869 | 491 | 26, 465 | IX |
| 2 | *Phlebotomus perfiliewi* | 2011-s | **Italy:** Matera | JF766965 | 21635869 | 480 | 26, 90, 104, 260 | X |
| 2 | *Phlebotomus perfiliewi* | 2011-s | **Italy:** Matera | JF766966 | 21635869 | 480 | 26, 90, 104, 260 | X |
| 2 | *Phlebotomus perfiliewi* | 2011-s | **Italy:** Matera | JF766967 | 21635869 | 480 | 26, 90, 104, 260 | X |
| 2 | *Phlebotomus perfiliewi* | 2011-s | **Italy:** Matera | JF766968 | 21635869 | 480 | 26, 90, 104, 260 | X |
| 2 | *Phlebotomus perfiliewi* | 2011-s | **Italy:** Matera | JF766969 | 21635869 | 480 | 26, 90, 104, 260 | X |
| 2 | *Phlebotomus perfiliewi* | 2011-s | **Italy:** Matera | JF766970 | 21635869 | 480 | 26, 90, 104, 260 | X |
| 2 | *Phlebotomus perfiliewi* | 2011-s | **Italy:** Matera | JF766971 | 21635869 | 480 | 26, 90, 104, 260 | X |
| 2 | *Phlebotomus perfiliewi* | 2011-s | **Italy:** Matera | JF766972 | 21635869 | 480 | 26, 90, 104, 260 | X |
| 2 | *Phlebotomus perfiliewi* | 2013-s | **Greece:** Macedonia | KF680806 | 24140558 | 480 | 26, 90, 104, 260 | X |
| 2 | *Phlebotomus perfiliewi* | 2013-s | **Greece:** Macedonia | KF680807 | 24140558 | 480 | 26, 90, 104, 260 | X |
| 2 | *Phlebotomus perfiliewi* | 2013-s | **Greece:** Macedonia | KF680808 | 24140558 | 480 | 26, 90, 104, 260 | X |
| 2 | *Phlebotomus perfiliewi* | 2013-s | **Greece:** Macedonia | KF680809 | 24140558 | 480 | 26, 90, 104, 260 | X |
| 2 | *Phlebotomus perfiliewi* | 2013-s | **Italy:** Continental | KF680810 | 24140558 | 480 | 26, 90, 104, 260 | X |
| 2 | *Phlebotomus perfiliewi* | 2013-s | **Italy:** Continental | KF680811 | 24140558 | 480 | 26, 90, 104, 260 | X |
| 2 | *Phlebotomus perfiliewi* | 2013-s | **Italy:** Continental | KF680812 | 24140558 | 480 | 26, 90, 104, 260 | X |
| 2 | *Phlebotomus perfiliewi* | 2013-s | **Italy:** Continental | KF680813 | 24140558 | 480 | 26, 90, 104, 260 | X |
| 2 | *Phlebotomus perfiliewi* | 2013-s | **Italy:** Continental | KF680814 | 24140558 | 480 | 26, 90, 104, 260 | X |
| 2 | *Phlebotomus perfiliewi* | 2013-s | **Italy:** Sardinia | KF680815 | 24140558 | 480 | 26, 90, 104, 260 | X |
| 2 | *Phlebotomus perfiliewi* | 2013-s | **Italy:** Sardinia | KF680816 | 24140558 | 480 | 26, 90, 104, 260 | X |
| 2 | *Phlebotomus perfiliewi* | 2013-s | **Italy:** Sardinia | KF680817 | 24140558 | 480 | 26, 90, 104, 260 | X |
| 2 | *Phlebotomus perfiliewi* | 2013-s | **Italy:** Sardinia | KF680818 | 24140558 | 480 | 26, 90, 104, 260 | X |
| 2 | *Phlebotomus perfiliewi* | 2013-s | **Algeria:** | KF680819 | 24140558 | 480 | 26, 90, 104, 260 | X |
| 2 | *Phlebotomus perfiliewi* | 2013-s | **Algeria:** | KF680820 | 24140558 | 480 | 26, 90, 104, 260 | X |
| 2 | *Phlebotomus perfiliewi* | 2013-s | **Algeria:** | KF680821 | 24140558 | 480 | 26, 90, 104, 260 | X |
| 2 | *Phlebotomus perfiliewi* | 2013-s | **Algeria:** | KF680822 | 24140558 | 480 | 26, 90, 104, 260 | X |
| 2 | *Phlebotomus perfiliewi* | 1997-s | **Greece:** Corfu | U94477 | 9272439 | 480 | 26, 90, 104, 260 | X |
| 2 | *Phlebotomus riouxi* | 2006 | **Algeria:** Ghardaïa, Ghardaïa | EU935815 | 19202763 | 472 | 26, 446 | VII |
| 2 | *Phlebotomus riouxi* | 2006 | **Algeria:** Ghardaïa, Ghardaïa | EU935816 | 19202763 | 472 | 26, 446 | VII |
| 2 | *Phlebotomus riouxi* | 2006 | **Algeria:** Ghardaïa, Ghardaïa | EU935817 | 19202763 | 472 | 26, 446 | VII |
| 2 | *Phlebotomus riouxi* | 2006 | **Algeria:** Metlili, Ghardaïa | EU935818 | 19202763 | 472 | 26, 446 | VII |
| 2 | *Phlebotomus riouxi* | 2006 | **Algeria:** Metlili, Ghardaïa | EU935819 | 19202763 | 472 | 26, 446 | VII |
| 2 | *Phlebotomus riouxi* | 2006 | **Algeria:** Metlili, Ghardaïa | EU935820 | 19202763 | 472 | 26, 446 | VII |
| 2 | *Phlebotomus riouxi* | 2006 | **Algeria:** Metlili, Ghardaïa | EU935821 | 19202763 | 472 | 26, 446 | VII |
| 2 | *Phlebotomus riouxi* | 2006 | **Algeria:** Metlili, Ghardaïa | EU935822 | 19202763 | 472 | 26, 446 | VII |
| 2 | *Phlebotomus riouxi* | 2006 | **Algeria:** Metlili, Ghardaïa | EU935823 | 19202763 | 472 | 26, 446 | VII |
| 2 | *Phlebotomus riouxi* | 2007 | **Tunisia:** Ghomrassen, Tataouine | EU935824 | 19202763 | 472 | 26, 446 | VII |
| 2 | *Phlebotomus riouxi* | 2007 | **Tunisia:** Ghomrassen, Tataouine | EU935825 | 19202763 | 472 | 26, 446 | VII |
| 2 | *Phlebotomus riouxi* | 2007 | **Tunisia:** Ghomrassen, Tataouine | EU935826 | 19202763 | 472 | 26, 446 | VII |
| 2 | *Phlebotomus riouxi* | 2007 | **Tunisia:** Ghomrassen, Tataouine | EU935827 | 19202763 | 472 | 26, 446 | VII |
| 2 | *Phlebotomus sergenti* | 2005 | **Iran:** Kerman, Kerman | DQ840374 | 17451632 | 476 | 26, 450 | XI |
| 2 | *Phlebotomus sergenti* | 2005 | **Iran:** Bam, Kerman | DQ840377 | 17451632 | 476 | 26, 450 | XI |
| 2 | *Phlebotomus sergenti* | 2011-s | **Tunisia:** Tataouine | JN036762 | 22897038 | 476 | 26, 206, 244 | XII |
| 2 | *Phlebotomus sergenti* | 2011-s | **Tunisia:** Tataouine | JN036764 | 22897038 | 476 | 26, 206, 244 | XII |
| 2 | *Phlebotomus sergenti* | 2013-s | **Tunisia:** | KF668645 | Unpublished | 476 | 26, 206, 244 | XII |
| 2 | *Phlebotomus sergenti* | 2005 | **Iran:** Sabzevar, Khorassan | DQ840348 | 17451632 | 476 | 26, 450 | XI |
| 2 | *Phlebotomus sergenti* | 2005 | **Iran:** Sabzevar, Khorassan | DQ840349 | 17451632 | 476 | 26, 450 | XI |
| 2 | *Phlebotomus sergenti* | 2005 | **Iran:** Sabzevar, Khorassan | DQ840350 | 17451632 | 476 | 26, 450 | XI |
| 2 | *Phlebotomus sergenti* | 2005 | **Iran:** Sabzevar, Khorassan | DQ840351 | 17451632 | 476 | 26, 450 | XI |
| 2 | *Phlebotomus sergenti* | 2005 | **Iran:** Neishabur, Khorassan | DQ840352 | 17451632 | 476 | 26, 450 | XI |
| 2 | *Phlebotomus sergenti* | 2005 | **Iran:** Neishabur, Khorassan | DQ840353 | 17451632 | 476 | 26, 450 | XI |
| 2 | *Phlebotomus sergenti* | 2005 | **Iran:** Neishabur, Khorassan | DQ840354 | 17451632 | 476 | 26, 450 | XI |
| 2 | *Phlebotomus sergenti* | 2003, 2004 | **Iran:** Tabriz, Eastern-Azerbaijan | DQ840357 | 17451632 | 476 | 26, 450 | XI |
| 2 | *Phlebotomus sergenti* | 2003, 2004 | **Iran:** Tabriz, Eastern-Azerbaijan | DQ840358 | 17451632 | 476 | 26, 450 | XI |
| 2 | *Phlebotomus sergenti* | 2003, 2004 | **Iran:** Tabriz, Eastern-Azerbaijan | DQ840359 | 17451632 | 476 | 26, 450 | XI |
| 2 | *Phlebotomus sergenti* | 2003, 2004 | **Iran:** Tabriz, Eastern-Azerbaijan | DQ840360 | 17451632 | 476 | 26, 450 | XI |
| 2 | *Phlebotomus sergenti* | 2004 | **Iran:** Meshgin-Shahr, Ardebil | DQ840361 | 17451632 | 476 | 26, 450 | XI |
| 2 | *Phlebotomus sergenti* | 2004 | **Iran:** Meshgin-Shahr, Ardebil | DQ840362 | 17451632 | 476 | 26, 450 | XI |
| 2 | *Phlebotomus sergenti* | 2003, 2005 | **Iran:** Tehran, Tehran | DQ840363 | 17451632 | 476 | 26, 450 | XI |
| 2 | *Phlebotomus sergenti* | 2003, 2005 | **Iran:** Tehran, Tehran | DQ840364 | 17451632 | 476 | 26, 450 | XI |
| 2 | *Phlebotomus sergenti* | 2003, 2005 | **Iran:** Tehran, Tehran | DQ840365 | 17451632 | 476 | 26, 450 | XI |
| 2 | *Phlebotomus sergenti* | 2003, 2005 | **Iran:** Tehran, Tehran | DQ840366 | 17451632 | 476 | 26, 450 | XI |
| 2 | *Phlebotomus sergenti* | 2003, 2005 | **Iran:** Tehran, Tehran | DQ840367 | 17451632 | 476 | 26, 450 | XI |
| 2 | *Phlebotomus sergenti* | 2005 | **Iran:** Kerman, Kerman | DQ840368 | 17451632 | 476 | 26, 450 | XI |
| 2 | *Phlebotomus sergenti* | 2005 | **Iran:** Kerman, Kerman | DQ840369 | 17451632 | 476 | 26, 450 | XI |
| 2 | *Phlebotomus sergenti* | 2005 | **Iran:** Kerman, Kerman | DQ840370 | 17451632 | 476 | 26, 450 | XI |
| 2 | *Phlebotomus sergenti* | 2005 | **Iran:** Kerman, Kerman | DQ840371 | 17451632 | 476 | 26, 450 | XI |
| 2 | *Phlebotomus sergenti* | 2005 | **Iran:** Kerman, Kerman | DQ840372 | 17451632 | 476 | 26, 450 | XI |
| 2 | *Phlebotomus sergenti* | 2005 | **Iran:** Kerman, Kerman | DQ840373 | 17451632 | 476 | 26, 450 | XI |
| 2 | *Phlebotomus sergenti* | 2005 | **Iran:** Bam, Kerman | DQ840375 | 17451632 | 476 | 26, 450 | XI |
| 2 | *Phlebotomus sergenti* | 2005 | **Iran:** Bam, Kerman | DQ840376 | 17451632 | 476 | 26, 450 | XI |
| 2 | *Phlebotomus sergenti* | 2005 | **Iran:** Bam, Kerman | DQ840378 | 17451632 | 476 | 26, 450 | XI |
| 2 | *Phlebotomus sergenti* | 2005 | **Iran:** Bam, Kerman | DQ840379 | 17451632 | 476 | 26, 450 | XI |
| 2 | *Phlebotomus sergenti* | 2005 | **Iran:** Yazd, Yazd | DQ840380 | 17451632 | 476 | 26, 450 | XI |
| 2 | *Phlebotomus sergenti* | 2005 | **Iran:** Yazd, Yazd | DQ840381 | 17451632 | 476 | 26, 450 | XI |
| 2 | *Phlebotomus sergenti* | 2005 | **Iran:** Yazd, Yazd | DQ840382 | 17451632 | 476 | 26, 450 | XI |
| 2 | *Phlebotomus sergenti* | 2005 | **Iran:** Yazd, Yazd | DQ840383 | 17451632 | 476 | 26, 450 | XI |
| 2 | *Phlebotomus sergenti* | 2005 | **Iran:** Izeh, Khuzistan | DQ840384 | 17451632 | 476 | 26, 450 | XI |
| 2 | *Phlebotomus sergenti* | 2005 | **Iran:** Izeh, Khuzistan | DQ840385 | 17451632 | 476 | 26, 450 | XI |
| 2 | *Phlebotomus sergenti* | 2005 | **Iran:** Izeh, Khuzistan | DQ840386 | 17451632 | 476 | 26, 450 | XI |
| 2 | *Phlebotomus sergenti* | 2003 | **Iran:** Shiraz, Fars | DQ840387 | 17451632 | 476 | 26, 450 | XI |
| 2 | *Phlebotomus sergenti* | 2003 | **Iran:** Shiraz, Fars | DQ840388 | 17451632 | 476 | 26, 450 | XI |
| 2 | *Phlebotomus sergenti* | 2005 | **Iran:** Bushehr | DQ840389 | 17451632 | 476 | 26, 450 | XI |
| 2 | *Phlebotomus sergenti* | 2005 | **Iran:** Bushehr | DQ840390 | 17451632 | 476 | 26, 450 | XI |
| 2 | *Phlebotomus sergenti* | 2005 | **Iran:** Bushehr | DQ840391 | 17451632 | 476 | 26, 450 | XI |
| 2 | *Phlebotomus sergenti* | 2005 | **Iran:** Bushehr | DQ840392 | 17451632 | 476 | 26, 450 | XI |
| 2 | *Phlebotomus sergenti* | 2005 | **Iran:** Bushehr | DQ840393 | 17451632 | 476 | 26, 450 | XI |
| 2 | *Phlebotomus sergenti* | 2005 | **Iran:** Bandar-Abbas, Hormozgan | DQ840394 | 17451632 | 476 | 26, 450 | XI |
| 2 | *Phlebotomus sergenti* | 2005 | **Iran:** Bandar-Abbas, Hormozgan | DQ840395 | 17451632 | 476 | 26, 450 | XI |
| 2 | *Phlebotomus sergenti* | 2003 | **Iran:** Angoori, Sistan & Baluchistan | DQ840396 | 17451632 | 476 | 26, 450 | XI |
| 2 | *Phlebotomus sergenti* | 2006-s | **Grece:** | DQ840398 | 17451632 | 476 | 26, 450 | XI |
| 2 | *Phlebotomus sergenti* | 2006-s | **Syria:** | DQ840401 | 17451632 | 476 | 26, 450 | XI |
| 2 | *Phlebotomus sergenti* | 2006-s | **Lebanon:** | DQ840402 | 17451632 | 476 | 26, 450 | XI |
| 2 | *Phlebotomus sergenti* | 2006-s | **Turkey:** | DQ840403 | 17451632 | 476 | 26, 450 | XI |
| 2 | *Phlebotomus sergenti* | 2006-s | **Morocco:** | DQ840404 | 17451632 | 476 | 26, 206, 244 | XII |
| 2 | *Phlebotomus sergenti* | 2004 | **Iran:** Tabriz, Eastern-Azerbaijan | DQ840405 | 17451632 | 476 | 26, 450 | XI |
| 2 | *Phlebotomus sergenti^* | 2011-s | **Tunisia:** Tataouine | JN036763 | 22897038 | 476 | 26, 206, 244 | XII |
| 2 | *Phlebotomus chadlii* | 2010-s | **Algeria:** Aurès | HM131080 | 20451525 | 477 | 26, 451 | XIII |
| 2 | *Phlebotomus chadlii* | 2010-s | **Algeria:** Aurès | HM131079 | 20451525 | 477 | 26, 451 | XIII |
| 2 | *Phlebotomus balcanicus* | 2012-s | **Iran?:** | JX885989-unverified | Unpublished | 472 | 26, 446 | VII |
| 2 | *Phlebotomus balcanicus* | 2012-s | **Iran?:** | JX885983-unverified | Unpublished | 472 | 26, 446 | VII |
| 2 | *Phlebotomus caucasicus* | 2005 | **Iran:** Neishabur, Khorassan-e-Razavi | EF017370 | 17982863 | 476 | 26, 214, 236 | XIV |
| 2 | *Phlebotomus caucasicus* | 2005 | **Iran:** Isfahan, Isfahan | EF017353 | 17982863 | 476 | 26, 214, 236 | XIV |
| 2 | *Phlebotomus caucasicus* | 2005 | **Iran:** Isfahan, Isfahan | EF017354 | 17982863 | 476 | 26, 214, 236 | XIV |
| 2 | *Phlebotomus caucasicus* | 2005 | **Iran:** Urmia, Western-Azerbaijan | EF017357 | 17982863 | 476 | 26, 214, 236 | XIV |
| 2 | *Phlebotomus caucasicus* | 2004 | **Iran:** MeshKin-shahr, Ardebil | EF017359 | 17982863 | 476 | 26, 214, 236 | XIV |
| 2 | *Phlebotomus caucasicus* | 2005 | **Iran:** Isfahan, Isfahan | EF017361 | 17982863 | 476 | 26, 214, 236 | XIV |
| 2 | *Phlebotomus caucasicus* | 2005 | **Iran:** Isfahan, Isfahan | EF017362 | 17982863 | 476 | 26, 214, 236 | XIV |
| 2 | *Phlebotomus caucasicus* | 2005 | **Iran:** Urmia, Western-Azerbaijan | EF017349 | 17982863 | 476 | 26, 214, 236 | XIV |
| 2 | *Phlebotomus caucasicus* | 2005 | **Iran:** Yazd, Yazd | EF017371 | 17982863 | 476 | 26, 214, 236 | XIV |
| 2 | *Phlebotomus caucasicus* | 2005 | **Iran:** Sabzevar, Khorassan-e-Razavi | EF017369 | 17982863 | 476 | 26, 214, 236 | XIV |
| 2 | *Phlebotomus caucasicus* | 2005 | **Iran:** Tabriz, Eastern-Azerbaijan | EF017368 | 17982863 | 476 | 26, 214, 236 | XIV |
| 2 | *Phlebotomus caucasicus* | 2005 | **Iran:** Yazd, Yazd | EF017367 | 17982863 | 476 | 26, 214, 236 | XIV |
| 2 | *Phlebotomus caucasicus* | 2005 | **Iran:** Yazd, Yazd | EF017358 | 17982863 | 476 | 26, 214, 236 | XIV |
| 2 | *Phlebotomus caucasicus* | 2005 | **Iran:** Kerman, Kerman | EF017365 | 17982863 | 476 | 26, 214, 236 | XIV |
| 2 | *Phlebotomus caucasicus* | 2005 | **Iran:** Neishabur, Khorassan-e-Razavi | EF017364 | 17982863 | 476 | 26, 214, 236 | XIV |
| 2 | *Phlebotomus caucasicus* | 2005 | **Iran:** Sabzevar, Khorassan-e-Razavi | EF017360 | 17982863 | 476 | 26, 214, 236 | XIV |
| 2 | *Phlebotomus caucasicus* | 2005 | **Iran:** Neishabur, Khorassan-e-Razavi | EF017355 | 17982863 | 476 | 26, 214, 236 | XIV |
| 2 | *Phlebotomus caucasicus* | 2005 | **Iran:** Neishabur, Khorassan-e-Razavi | EF017363 | 17982863 | 476 | 26, 214, 236 | XIV |
| 2 | *Phlebotomus caucasicus* | 2005 | **Iran:** Kerman, Kerman | EF017352 | 17982863 | 476 | 26, 214, 236 | XIV |
| 2 | *Phlebotomus caucasicus* | 2005 | **Iran:** Sabzevar, Khorassan-e-Razavi | EF017351 | 17982863 | 476 | 26, 214, 236 | XIV |
| 2 | *Phlebotomus caucasicus* | 2009 | **Afghanistan:** Kabul | HM803207 | 21396252 | 476 | 26, 214, 236 | XIV |
| 2 | *Phlebotomus caucasicus* | 2009 | **Afghanistan:** Kondoz | HM803215 | 21396252 | 476 | 26, 214, 236 | XIV |
| 2 | *Phlebotomus caucasicus* | 2005 | **Iran:** Kerman, Kerman | EF017356 | 17982863 | 476 | 26, 214, 236 | XIV |
| 2 | *Phlebotomus caucasicus* | 2009 | **Afghanistan:** MeS | HM803213 | 21396252 | 476 | 26, 214, 236 | XIV |
| 2 | *Phlebotomus caucasicus* | 2009 | **Afghanistan:** Ghowrmach | HM803206 | 21396252 | 476 | 26, 450 | XI |
| 2 | *Sergentomyia minuta* | 2008, 2009 | **Italy: Putignano, Bari** | JF766976 | 21635869 | 482 | 17, 26, 72, 131, 236 | XVI |
| 2 | *Sergentomyia minuta* | 2008, 2009 | **Italy: Putignano, Bari** | JF766977 | 21635869 | 482 | 17, 26, 72, 131, 236 | XVI |
| 2 | *Sergentomyia minuta* | 2008, 2009 | **Italy: Putignano, Bari** | JF766978 | 21635869 | 482 | 17, 26, 72, 131, 236 | XVI |
| 2 | *Sergentomyia minuta* | 2008, 2009 | **Italy: Putignano, Bari** | JF766979 | 21635869 | 482 | 17, 26, 72, 131, 236 | XVI |
| 2 | *Sergentomyia minuta* | 2008, 2009 | **Italy: Putignano, Bari** | JF766980 | 21635869 | 482 | 17, 26, 72, 131, 236 | XVI |
| 2 | *Sergentomyia minuta* | 2008, 2009 | **Italy: Putignano, Bari** | JF766981 | 21635869 | 482 | 17, 26, 40, 72, 91, 236 | XV |

yyyy-s: indicates date of submission to GenBank, in these sequences it was not possible to accurately identify the collection year

*: though the paper is referred in the GenBank Acc No data, it is not clear that the sequences are related to the journal publication.

?: indicates the possible location, however this is not completely clear according to the information provided either in the GenBank Acc No or the journal publication related to it.

^P. longicuspis in the publication, but appears as P. sergenti in GenBank

**Reference as PubMed, unless otherwise indicated
