# Supplementary figures and images for: DNA sequence analysis suggests that cytb-nd1 PCR-RFLP may not be applicable to sandfly species identification throughout the Mediterranean region
Source: Parasitol Res. 2016 Jan 12;115:1287–95. doi: 10.1007/s00436-015-4865-5 (PMC4759228; doi:10.1007/s00436-015-4865-5)

**Supplementary material 2**
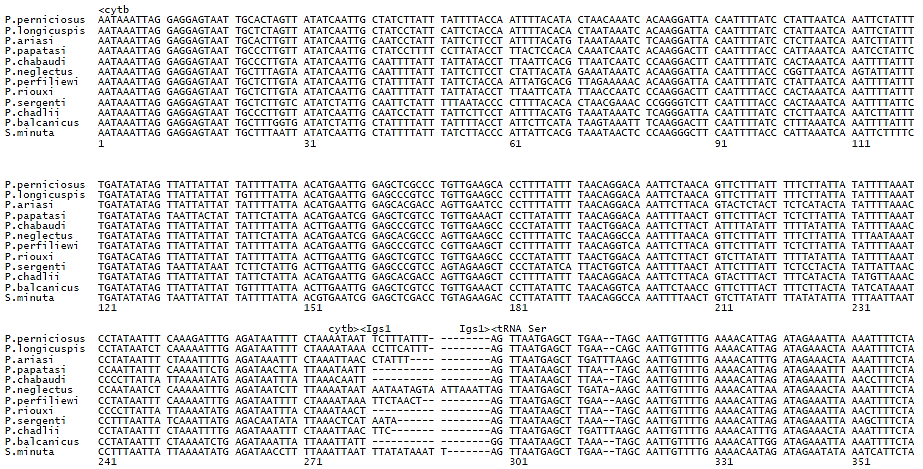

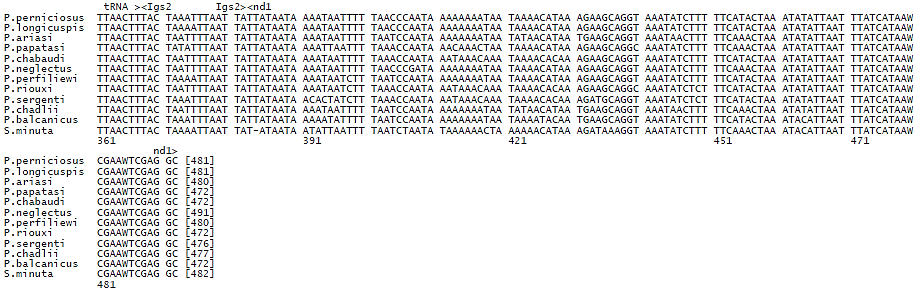

Supplement: Supplementary file 2 — Nucleotide alignment of the cytb-nd1 DNA sequences from representative specimens for each phlebotomine sandfly species studied in this work. The corresponding genes are reported on top of the alignment. Numbers at the bottom indicate nucleotide positions. Numbers at the end of each line indicate the length (in bp) of each sequence. (DOCX 797 kb) [file 436_2015_4865_MOESM2_ESM.docx]

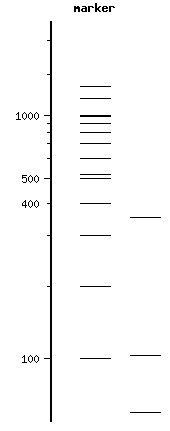

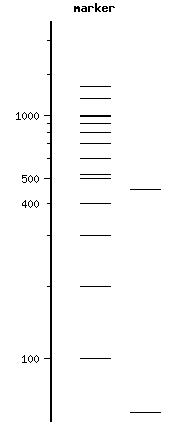

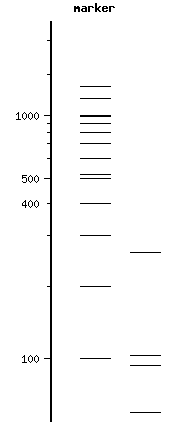

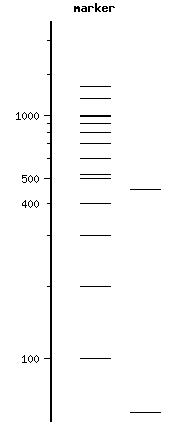

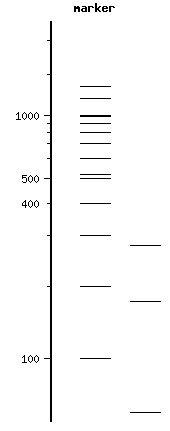

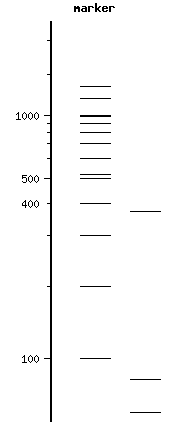

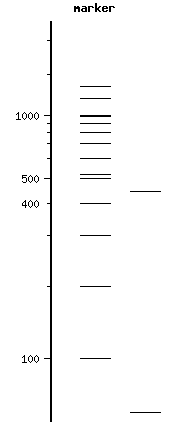

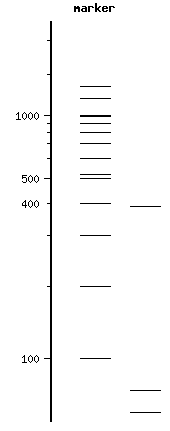

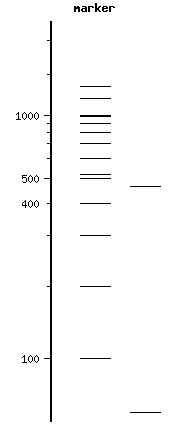

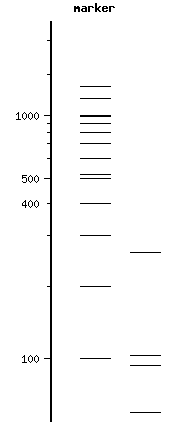

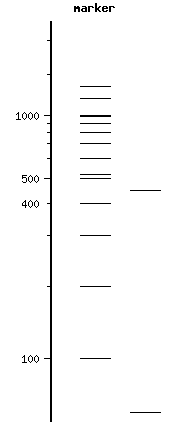

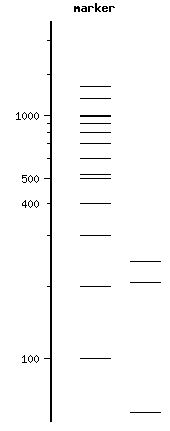

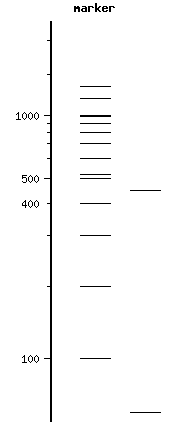

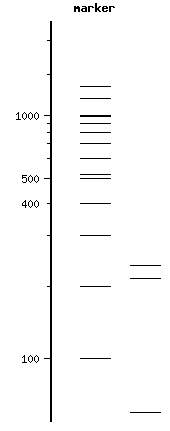

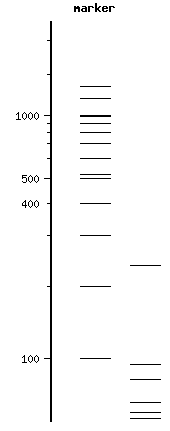

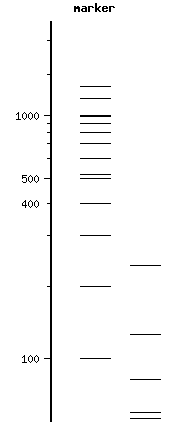


I II III IV V VI VII VIII IX X XI XII XIII XIV XV XVI

**Supplementary material 3**

Supplement: Supplementary file 3 — Virtual agarose gel produced with NEBcutter V2.0 showing the sixteen different RFLP patterns obtained from the cytb-nd1 DNA sequences included in assemblies 1 and 2. Patterns are indicated in Roman numerals. The virtual run shows the digested products on a theoretical 2% agarose gel electrophoresis and the full resolution of a 100 bp ladder molecular weight marker, L=70mm. Pattern I: P. perniciosus_1, P. longicuspis_2; Pattern II: P. perniciosus_2; Pattern III: P. longicuspis_1; Pattern IV: P. ariasi_1; Pattern V: P. ariasi_2; Pattern VI: P. papatasi; Pattern VII: P. riouxi, P. balcanicus, P. chabaudi_1; Pattern VIII: P. chabaud_2i; Pattern IX: P. neglectus; Pattern X: P. perfiliewi; Pattern XI: P. sergenti_1, P. caucasicus_1; Pattern XII: P. sergenti_2; Pattern XIII: P. chadlii; Pattern XIV: P. caucasicus_2; Pattern XV: S. minuta_1; Pattern XVI: S. minuta_2. (DOCX 217 kb) [file 436_2015_4865_MOESM3_ESM.docx]
